# Single cell transcriptome atlas of mouse mammary epithelial cells across development

**DOI:** 10.1186/s13058-021-01445-4

**Published:** 2021-06-29

**Authors:** Bhupinder Pal, Yunshun Chen, Michael J. G. Milevskiy, François Vaillant, Lexie Prokopuk, Caleb A. Dawson, Bianca D. Capaldo, Xiaoyu Song, Felicity Jackling, Paul Timpson, Geoffrey J. Lindeman, Gordon K. Smyth, Jane E. Visvader

**Affiliations:** 1 ACRF Cancer Biology and Stem Cells Division, The Walter and Eliza Hall Institute of Medical Research, Parkville, VIC 3052 Australia; 2 Department of Medical Biology, The University of Melbourne, Parkville, VIC 3010 Australia; 3 School of Cancer Medicine, La Trobe University, Bundoora, VIC 3086 Australia; 4 Olivia Newton-John Cancer Research Institute, Heidelberg, VIC 3084 Australia; 5 Bioinformatics Division, The Walter and Eliza Hall Institute of Medical Research, Parkville, VIC 3052 Australia; 6 Cancer Division, Garvan Institute of Medical Research and Kinghorn Cancer Centre, University of NSW, Sydney, NSW 2010 Australia; 7 Department of Medicine, The University of Melbourne, Parkville, VIC 3010 Australia; 8 Parkville Familial Cancer Centre and Department of Medical Oncology, The Royal Melbourne Hospital and Peter MacCallum Cancer Centre, Parkville, VIC 3052 Australia; 9 School of Mathematics and Statistics, The University of Melbourne, Parkville, VIC 3010 Australia

**Keywords:** Single cell transcriptome, Molecular heterogeneity, Mammary gland development, Progenitors, Terminal end bud, Chromatin accessibility

## Abstract

**Background:**

Heterogeneity within the mouse mammary epithelium and potential lineage relationships have been recently explored by single-cell RNA profiling. To further understand how cellular diversity changes during mammary ontogeny, we profiled single cells from nine different developmental stages spanning late embryogenesis, early postnatal, prepuberty, adult, mid-pregnancy, late-pregnancy, and post-involution, as well as the transcriptomes of micro-dissected terminal end buds (TEBs) and subtending ducts during puberty.

**Methods:**

The single cell transcriptomes of 132,599 mammary epithelial cells from 9 different developmental stages were determined on the 10x Genomics Chromium platform, and integrative analyses were performed to compare specific time points.

**Results:**

The mammary rudiment at E18.5 closely aligned with the basal lineage, while prepubertal epithelial cells exhibited lineage segregation but to a less differentiated state than their adult counterparts. Comparison of micro-dissected TEBs versus ducts showed that luminal cells within TEBs harbored intermediate expression profiles. Ductal basal cells exhibited increased chromatin accessibility of luminal genes compared to their TEB counterparts suggesting that lineage-specific chromatin is established within the subtending ducts during puberty. An integrative analysis of five stages spanning the pregnancy cycle revealed distinct stage-specific profiles and the presence of cycling basal, mixed-lineage, and 'late' alveolar intermediates in pregnancy. Moreover, a number of intermediates were uncovered along the basal-luminal progenitor cell axis, suggesting a continuum of alveolar-restricted progenitor states.

**Conclusions:**

This extended single cell transcriptome atlas of mouse mammary epithelial cells provides the most complete coverage for mammary epithelial cells during morphogenesis to date. Together with chromatin accessibility analysis of TEB structures, it represents a valuable framework for understanding developmental decisions within the mouse mammary gland.

**Supplementary Information:**

The online version contains supplementary material available at 10.1186/s13058-021-01445-4.

## Introduction

The mammary epithelium undergoes remarkable remodeling during the different stages of postnatal morphogenesis [1, 2]. During embryonic development, mammary morphogenesis initiates from the ectoderm at E10.5, giving rise to 5 pairs of placodes by E11.5. At E13.5, a bud forms via invagination of the mesenchyme; these buds sprout by E15.5 and form a lumen, and by E18.5, a small arborized ductal structure has invaded the mammary fat pad. The majority of development, however, occurs in the postnatal animal. During puberty, the ductal tree elongates and bifurcates to form an extensive ductal network that fills the mammary fat pad. This process is driven by terminal end buds (TEBs), which are located at the termini of the growing ducts [3]. Pregnancy is accompanied by the prolific expansion of alveoli that emanate from the branches and constitute the milk-producing units to enable lactation. The final process of involution entails large-scale cell death of alveolar units and remodeling of the gland to its prepregnant state [4]. Structurally, the epithelium of the ductal tree is bilayered and comprises an inner layer of luminal cells and an outer layer of myoepithelial cells that directly contact the underlying basement membrane.

Recent transcriptional mapping studies at the single cell level have shed light on heterogeneity within the different epithelial populations in the mouse mammary gland and potential spatio-temporal relationships [5–9]. Analysis of the adult gland revealed three major epithelial subsets corresponding to the basal, luminal progenitor (LP; also referred to as secretory), and mature luminal (ML; also referred to as hormone-responsive) populations, consistent with cell sorting studies. Equivalent populations have been observed in human breast epithelium by single cell transcriptomics [10]. Single cell (sc) RNA-seq analysis of epithelial cells isolated during pregnancy has uncovered uni-lineage clusters [5] as well as a mixed-lineage subset [9]. Lineage specification has been inferred to occur after birth, based on scRNA-seq analysis of the early developing mouse mammary gland [6]. While chromatin accessibility analysis has indicated multilineage potential in both fetal and adult basal/myoepithelial mammary cells [11], recent single-nucleus ATAC-seq profiling revealed the presence of cells with either luminal or basal-oriented chromatin structure before birth [12]. The precise timing of lineage commitment, however, remains an area of investigation. Further chromatin accessibility analysis of the mouse adult mammary gland has indicated that LP or secretory cells can be divided into different states [13].

To build on previous scRNA-seq studies in the mammary gland field, we interrogated the transcriptomes of > 132,000 individual cells across different developmental stages. We incorporated a number of additional stages for analysis on the 10x Chromium platform: prepuberty, 12.5 and 18.5 days of pregnancy, post-involution, micro-dissected TEB and subtending ductal structures, as well as the resting adult gland from different mouse strains. The findings point to a dynamic molecular landscape whereby unique but basal-like embryonic mammary cells commence lineage segregation in the early postnatal period (prior to 2 weeks), yielding lineage-restricted cells that overlap with but are not identical to those in the adult. In puberty, marked differences in chromatin accessibility and the expression profiles of TEB- vs duct-enriched epithelia were found, while an alveolar-like intermediate was identified in late pregnancy. Together, these data provide a comprehensive single cell transcriptome resource for the mouse mammary gland across development and a detailed molecular characterization of ductal versus TEB structures in puberty.

## Methods

### Mouse strains

FVB/N, C57BL/6, and SWISS mice (WEHI animal facility) were used for the preparation of mammary epithelial cell suspensions for single-cell or bulk RNA-seq work. *Lgr5-GFP* [14] and *E-cadherin-GFP* mice [15] were generated as described. All animal experiments conformed to regulatory standards and were approved by the Walter and Eliza Hall Institute Animal Ethics Committee.

### Mammary cell preparation and cell sorting

Mammary rudiments and skin from C57BL/6 *Lgr5-GFP*+ embryos at E18.5 (n = 49 mammary rudiments from 7 female embryos isolated from three pregnant C57BL/6 females) were dissected using fluorescence microscopy and digested to generate single cell suspensions for staining and flow cytometry. TEBs and ducts were dissected from 5 week-old mammary glands obtained from *Ecadherin-GFP* females (C57BL/6), using GFP to guide microdissection of the TEBs using a Leica Fluorescence stereo-microscope (Leica Microsystems GmbH), then single-cell suspensions were prepared and stained for flow cytometry. C57BL/6 mice were used to collect mammary glands at day 5 after birth or at 2 weeks (prepuberty; days 15–17). For analysis of adult mammary epithelial cells, 10-week-old glands were collected from FVB/N, SWISS, and C57BL/6 mice. For the pregnancy cycle analysis (FVB/N mice), the following mammary glands were collected: virgin adult (10 weeks), pregnancy (days 12.5 and 18.5), lactation (day 10), and post-involution (day 21). Mammary glands from 3 to 4 mice were pooled for each time point.

Single-cell suspensions were prepared and stained for flow cytometry as described [16]. The following antibodies were used for FACS: FITC anti-mouse CD29 (rat, clone HMβ1-1, BioLegend Cat#102206, 1/200 dilution), Pacific Blue anti-mouse CD29 (rat, clone HMβ1-1, BioLegend Cat#102224, 1/200 dilution), Pacific Blue anti-mouse CD24 (Armenian Hamster, clone M1/69, BioLegend Cat#101820, 1/200), PE anti-mouse CD24 (Armenian Hamster, clone M1/69, BioLegend Cat#10807, 1/200), APC anti-mouse CD31 (rat, clone 390, BioLegend Cat#102410, 1/40 dilution), APC anti-mouse CD45 (rat, clone 30-F-11, BioLegend, Cat#103112, 1/100 dilution), and APC anti-mouse TER-119/erythroid cell (rat, clone TER-119, BioLegend Cat#116212, 1/100 dilution). To exclude dead cells, cells were re-suspended in 7-AAD prior to analysis. FACS analysis and cell sorting were performed on a FACS Aria (Becton Dickinson). The Lin^–^ population was defined as Ter119^–^CD31^–^CD45^–^. FACS data were analyzed using FlowJo software (v 10.1r7, Tree Star).

### Confocal immunofluorescence of mammary gland sections

Tissue sections were incubated overnight at 4^°^C with primary antibodies: Keratin 8/K18 (rat, clone: TROMA-I, DSHB, 1:400 dilution), MYLK (rabbit, clone: EP1458Y, Abcam, 1:200 dilution), and MYH11 (rabbit, polyclonal, ab53219, Abcam, 1:100 dilution). The following day, sections were incubated with secondary antibodies: donkey anti-rat Alexa Fluor 488 (Invitrogen; 1:500 dilution), donkey anti-rabbit Alexa Fluor 647 (Invitrogen; 1:500 dilution), and DAPI (62248, Thermo Scientific, 1:500 dilution). Confocal imaging was performed using a Zeiss LSM780 or 980 microscope and images were processed in Imaris.

### Single cell capture and NGS library preparation for sequencing

For high-throughput single cell studies, the 10X Genomics Chromium kit (v2) was used for single cell capture and cDNA preparation according to the 10x Single Cell 3’ Protocol. Freshly sorted cells were manually counted and equal numbers per sample (1000 cells/μl) were loaded for capture, except in the case of the TEB/duct and postnatal day 5 samples for which cell numbers were very limiting. The following samples were processed concurrently: E18.5 skin and ME; TEBs and ducts; 2-week-old C57BL/6, adult C57BL/6, and adult SWISS mammary glands; and FVB/N adult and post-involution. The final libraries contain the P5 and P7 primers used in Illumina bridge amplification. Sequencing was carried out on an Illumina Nextseq 500 with a maximum of 2 libraries per run.

### scRNA-seq bioinformatic analysis

Illumina output from 10X Genomics Chromium sequencing reads was processed using Cell Ranger v3.0.2. Genewise read counts for cells with at least 500 reads were exported to Matrix Market format files and read into R with edgeR’s read10X function. An average of > 55 million reads and > 8500 cells were obtained for each sample (Table S1). Cells with less than 500 genes detected or with high mitochondrial percentages were filtered. Cells with exceptionally high numbers of reads or genes detected were also filtered to minimize the occurrence of doublets. An average of 7500 cells per sample remained after this quality filtering (Table 1, Table S1). Statistical analyses of the 10X data were conducted using the Seurat (V3.1.5) [17] and edgeR [18] software packages. Gene symbols from Cell Ranger were converted to current official gene symbols using limma’s alias2SymbolUsingNCBI function and NCBI gene annotation dated 12 February 2019. Genes that did not map to official symbols were filtered as were genes expressed in < 1% cells for any individual sample. If two or more Cell Ranger genes mapped to the same official symbol, then the one with largest read count was kept for each sample.

**Table 1:** Summary of single cell RNA-seq datasets

Developmental phase	Number of epithelial cells
Embryonic (E18.5) mammary	6398
Embryonic (E18.5) adjacent skin	9025
Early postnatal (5 days)	3728
Pre-puberty	4037
Puberty (5 weeks)	
TEBs	1847
Ducts	1700
Adult	
FVB/N (A10) #1	10,710
FVB/N (P7) #2	3187
FVB/N (D12) #3	5576
C57BL/6	12,525
SWISS	10,319
Pregnancy	
12.5 days (H) #1	11,120
12.5 days (G) #2	13,397
18.5 days (F4) #1	11,847
18.5 days (B4) #2	5075
Lactation (10 days)	
10 days (F11) #1	5287
10 days (D6) #2	6804
Post-involution (3 weeks)	10,017
Total	132,599

Total epithelial cells were isolated from the stages listed above for capture and sequencing on the 10X Genomics Chromium platform. Cell numbers refer to those retained after filtering out cells with low sequencing reads. # denotes the replicate number

Where appropriate, multiple samples were combined using the anchor-based integration method implemented in Seurat. Cell clusters were identified using the default Louvain clustering algorithm implemented in Seurat. Default Seurat function settings were used except that clustering resolutions were set to lower than default values in order to ensure conservative and reproducible clusters and dimensions 1:20 were used for all dimension reduction and integration steps. The resolution was set to 0.1 unless stated otherwise. Marker genes of each cluster were identified using Seurat’s FindMarkers function with default settings. Ternary plots position cells according to the proportion of basal, LP, or ML positive signature genes expressed by that cell and were generated using the vcd package. Genes differentially expressed between TEB and Duct samples were identified using FindMarkers with logfc.threshold = 0.15 and otherwise default settings. Log2-CPM values for each gene across cells were calculated using edgeR’s cpm function with a prior count of 1. Heatmaps were generated using the pheatmap package. Log2-CPM values were standardized to have mean 0 and standard deviation 1 for each gene before producing the heatmaps, after which genes and cells were clustered by the Ward’s minimum variance method. Diffusion plots were generated using the destiny package.

### Signature genes for epithelial cell lineages

Signatures genes for basal, luminal progenitor (LP), and mature luminal (ML) cell types were obtained from previously published RNA-seq data [19] available from GEO series GSE63310. Only protein-coding genes were included. Genes were filtered if they failed to achieve 0.4 counts-per-million (CPM) in at least 3 samples. Library sizes were normalized by the TMM method in edgeR and expression analysis was conducted using limma voom [20]. Comparisons were performed using a TREAT fold-change threshold of 2 for basal vs LP and basal vs ML and 1.3 for LP vs ML [21]. Adjustment was made for sequencing lane as a blocking factor. An FDR cut-off of 0.05 was applied for each comparison. Genes were considered cell type-specific if they were upregulated in one cell type vs both other types. This analysis produced 1467, 428, and 528 signature genes for basal, LP, and ML respectively, which were used to construct ternary plots and lineage signature scores. Negative signature genes were also generated that were downregulated in one cell type vs both other types. This produced 1012, 188, and 226 negative signature genes for basal, LP and ML respectively.

### Bulk ATAC-seq of pubertal TEB and ductal regions

Mammary glands from 5-week-old *Ecad*-GFP expressing mice were micro-dissected for TEBs and ducts. Nuclei were isolated from sorted luminal (Lin^–^CD29^+^CD24^+^Ecad-GFP^hi^) and basal cells (Lin^–^CD29^+^CD24^+^Ecad-GFP^lo^), and ATAC-sequencing was performed as established by Buenrostro et al. [22]. Three biological replicates were prepared although one basal duct sample was discarded because of low cell numbers. For each sample, 15,000–25,000 sorted cells were lysed and transposition reactions were done in half volumes (25 μl lysis = 10 mM Tris-Cl (Tris-Cl pH 7.4), 10 mM NaCl, 3 mM MgCl_2_, 0.1% NP-40 in nuclease-free H_2_O) (25 μl transposition = 12.5 μl of 2X TD Buffer (Illumina Cat#FC-121-1030), 1.25 μl of TDE1 (Illumina Cat#FC-121-1030), 11.25 μl of nuclease-free H_2_O). Transposition was performed for 20 min at 22 °C then 30 min at 37 °C and purified with the MinElute kit from Qiagen (Cat#28204). Libraries were sequenced on an Illumina NextSeq using the 150H kit to produce > 10 million 80 bp paired-end sequence reads per sample. Adapter sequences were trimmed, reads were mapped to the mm10 genome with Bowtie2 v2.3.4.1 [23], and duplicate reads removed. Peaks were called with MACS2 v2.1.0 (FDR < 0.10) [24] on the pooled biological replicates, separately for each tissue and cell population*.* To establish a list of peaks for differential analysis, overlapping peaks were merged with bedtools v2.26.0 [25] and peaks overlapping with genomic blacklisted regions were subtracted. Read counts per peak in each sample were obtained in SeqMonk (v1.46.0, https://www.bioinformatics.babraham.ac.uk/projects/seqmonk/). For descriptive plots, peak intensities are represented as log2(RPKM+1). Differential accessibility analysis was conducted using the classic pipeline of edgeR [26]. For each pair of cell populations to be compared, library sizes were TMM normalized and exact tests conducted for each peak. Peaks with FDR < 0.05 were considered differentially accessible (DA). For comparison of signature gene chromatin accessibility between basal duct- and TEB-enriched populations, the lm function in R was used to perform linear modeling.

### Bulk RNA-seq of pubertal TEB and ductal regions

Mammary glands from 5-week-old *Ecad*-GFP expressing mice were micro-dissected for TEBs and ducts and luminal (Lin^–^CD29^+^CD24^+^Ecad-GFP^hi^), and basal cells (Lin^–^CD29^+^CD24^+^*Ecad*-GFP^lo^) were sorted by flow cytometry. Three biological replicates were prepared, with at least two mice pooled in each replicate, although one luminal duct sample was discarded because of low cell numbers. Total RNA (~ 10 ng) was used to generate libraries for whole transcriptome analysis following the Illumina’s TruSeq RNA v2 sample preparation protocol. Libraries were sequenced on an Illumina NextSeq 500 to produce 80 bp paired-end reads. Reads were aligned to the mm10 genome and counted by Entrez Genes using Rsubread and Rsubread’s inbuilt mm10 RefSeq gene annotation [27]. Between 30 and 50 million read-pairs were successfully assigned to genes. Gene annotation was downloaded from the NCBI (12 February 2019). Downstream analysis used the edgeR package [28]. Low expressed genes were filtered using filterByExpr, and library sizes were normalized by the TMM method. Log2-fold expression changes between TEBs and ducts or between luminal and basal populations were computed using edgeR’s estimateDisp and glmFit functions.

### Data availability

Sequence data have been deposited in the GEO database under accession code GSE164017 and GSE164307.

## Results

### Heterogeneity within the embryonic mammary epithelium and skin

**Fig. 1:**
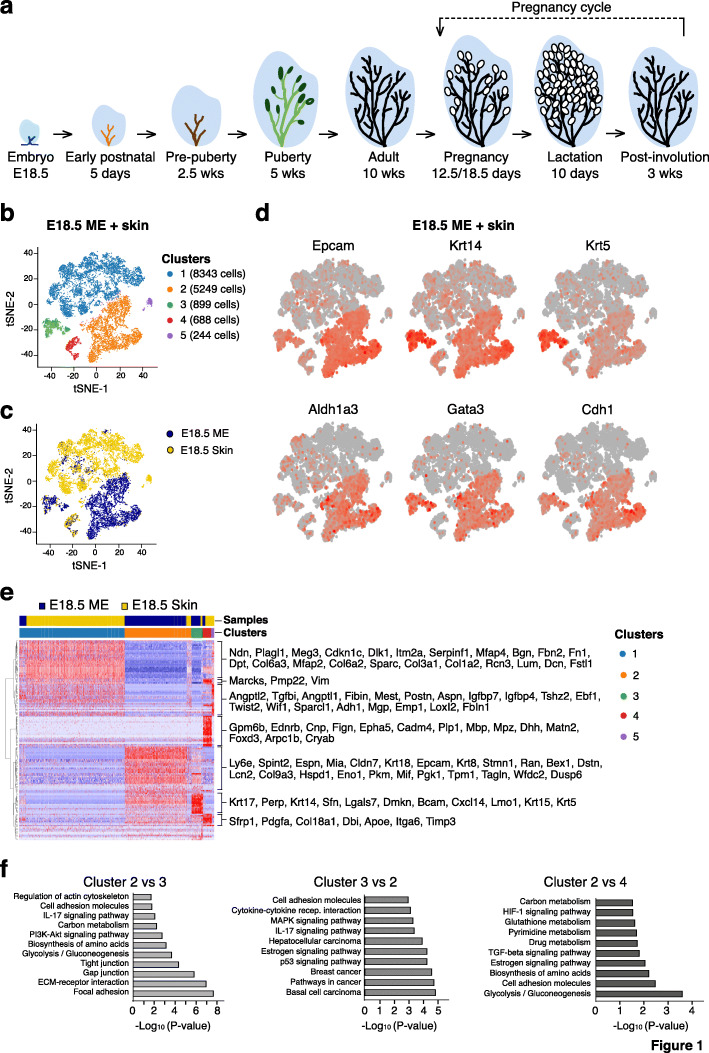
Heterogeneity among E18.5 mammary epithelium and adjacent skin cells. **a** Schematic diagram showing key developmental stages for the mouse mammary gland. **b** t-SNE plots showing the combined single cell transcriptomes of mammary epithelial cells and adjacent skin cells isolated from E18.5 embryos (n = 7 female embryos). Cell clusters identified by Seurat’s Louvain algorithm with resolution 0.03. **c** Same t-SNE plot as in **b** but colored by cell type: mammary epithelial cells (blue) and adjacent skin cells (yellow). **d** Same t-SNE plot as **b** and **c** but colored by relative expression (gray = low, red = high) of epithelial (*Epcam*), basal (*Krt14*, *Krt5*), and luminal (*Ecad*, *Aldh1a3*, and *Gata3*) genes. **e** Heat map showing relative log-expression of the top 30 marker genes for each cell cluster identified in Fig. 1b. **f** KEGG pathway analysis of genes differentially expressed between clusters shown in **b**

To construct an extended single cell atlas of the mammary gland, we profiled cells from late embryogenesis through to post-involution (Fig. 1a). The 10X Genomics Chromium platform was used to create scRNA-seq libraries for mammary epithelial cells across 9 developmental stages, yielding expression profiles for 132,599 cells after quality filtering (Table S1).

We began by addressing the molecular diversity among the embryonic mammary epithelium and adjoining epidermis at E18.5. Fluorescence from the *Lgr5-GFP-IRES-creERT2* knock-in mouse [14] was used to locate and dissect mammary rudiments. As the developing mammary gland is an ectodermal appendage closely associated with skin, we isolated adjacent skin to provide a control for epithelial purification. Lineage^–^ cells (depleted of endothelial and hematopoietic cells) were sorted by flow cytometry on the basis of CD24 and CD29 expression. Mammary epithelial cells expressed higher levels of CD24 (CD24^hi^) and *Lgr5-GFP* than the adjoining embryonic skin derivatives (Fig. S1A, B). Single cell transcriptomes were obtained for 6398 embryonic mammary epithelial (ME) cells and 9025 skin cells after quality control. To facilitate comparison of ME vs skin cells, the two single cell profiles were integrated using the Seurat pipeline [29]. Cell clustering of the integrated single cell profiles identified five clusters, as visualized by t-distributed stochastic neighbor embedding (t-SNE) dimension reduction in Fig. 1b. Marking cells according to tissue-origin underscored the distinct nature of each ectodermal tissue (Fig. 1c), with skin cells concentrated in clusters 1, 4, and 5 and ME cells in clusters 2 and 3.

Expression analysis of characteristic mammary epithelial genes (*Epcam*, *Krt14*, *Krt5*, *Aldh1a3*, *Gata3*, *Cdh1)* confirmed the mammary identity of clusters 2 and 3 (Fig. 1d). Hierarchical clustering based on the top expressed genes among the different clusters of the two tissues (Fig. 1e) revealed that in addition to mammary epithelial-specific genes (as above), cluster 2 expressed genes that were unique to the mammary rudiment (e.g., *Bex1/4*, *Dstn*, *Lcn2*, *Spint2*, *Espn*). Cluster 3 was characterized by strong expression of basal genes including *Krt5*, *Krt14*, *Krt17*, and *Cxcl14.* A single population, likely corresponding to cluster 2, was previously observed for Epcam^+^ primordial mammary cells at E18 [6]. For skin-derived cells, expression analysis indicated that the major cluster (#1) was enriched for fibroblast genes, while cluster 4 comprised cells that resembled fetal epidermal cells, which included expression of *Itga6* and *Epha5* (Fig. 1e). Conversely, low expression of embryonic skin/fibroblast genes was evident in the primordial mammary epithelial clusters (Fig. S1C). Some contamination of the ME cell preparation (blue) with adjacent epidermal cells is discernible in cluster 4 (Fig. 1c).

**Fig. 2:**
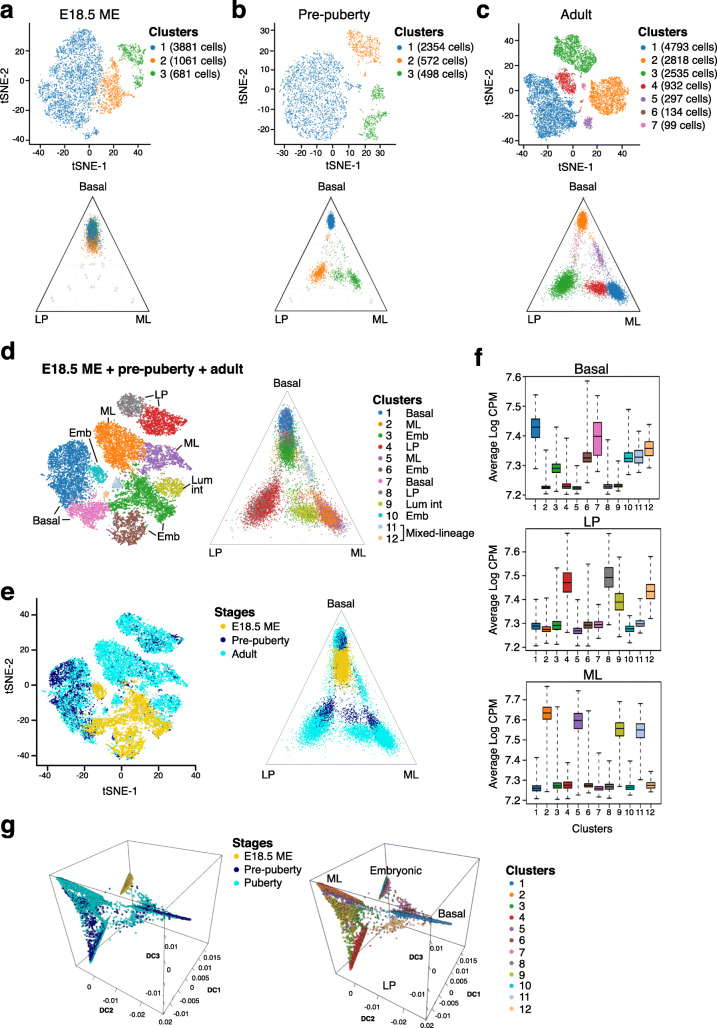
Molecular and cellular heterogeneity within the developing mammary epithelium. **a** t-SNE and ternary plots of C57BL/6-derived mammary epithelial cells from E18.5 embryos (n = 49 rudiments). Stromal contamination (see Fig. S2A) has been removed. Both plots show the same cells with the same cluster colors. In the ternary plot, each cell is positioned according to the proportion of basal, LP, or ML signature genes expressed by that cell. The three vertices of the plot correspond to cells expressing basal genes only, LP genes only, or ML genes only. Cells expressing equal numbers of basal, LP and ML genes are in the center of the plot. **b** Same as in **a** but for mammary epithelial cells from pre-pubertal C57BL/6 mice (2 weeks; n = 32 mice; cluster resolution 0.01). **c** Same as in **a** but for mammary epithelial cells from C57BL/6 adult mice (10 weeks; n = 5 mice; cluster resolution 0.2). **d** t-SNE and ternary plots of all cells from E18.5, pre-pubertal, and adult mice (as shown in **a**–**c**) after Seurat integration. Cells clustered with resolution 0.45. **e** Same plots as in **d** but colored according to developmental stage. **f** Box plots of lineage-specific expression signatures for the same cells and clusters as in **d**. Each epithelial cell was interrogated with the expression signatures for the human basal, LP, and ML cell types. Vertical axis shows average expression of cell population signature genes as log2 counts per million. **g** Diffusion maps of E18.5, pre-pubertal, and adult mammary glands cells according to developmental stage (left) and cell clusters (right)

KEGG pathway analysis of ME clusters 2 versus 3 indicated that cluster 2 was enriched for focal adhesion, ECM-receptor interactions, tight junction interactions, PI3-Akt and IL-17 signaling. Conversely, the basal-like cluster 3 (high *Krt5* and *Krt14*) was enriched for estrogen signaling, breast cancer, as well as the p53, MAPK, and cytokine signaling pathways (Fig. 1f). These cells may possibly represent “intermediates” between embryonic mammary cells and the postnatal basal compartment (see also cluster 10 in Fig. 2d). Interestingly, the major ME subset was enriched for diverse metabolic pathways (e.g., glycolysis, glutathione, pyrimidine) and TGFβ signaling compared to embryonic epidermal cells.

### Integrated analysis of embryonic, prepubertal, and adult mammary epithelial cells highlights the unique landscape of embryonic cells

To further understand the molecular changes that accompany postnatal development, we determined the single cell transcriptomes of sorted mammary epithelial cells (Lin^–^CD29^hi^CD24^+^) from prepubertal (2 weeks) C57BL/6 mice and compared them with those of E18.5 and adult mammary epithelial cells of the same strain. Cell clustering and expression analysis identified small clusters of contaminating skin or stromal cells (Fig. S2A, B), thus for subsequent analyses to probe epithelial cell relationships, the stromal clusters were removed and the remaining cells reclustered (Fig. 2a–c).

To display the lineage identity of the clusters, we used ternary plots to position each cell according to its expression of > 2400 known markers of the basal, LP, and ML epithelial subtypes [9, 19] (Fig. 2a–c lower panels). The ternary plot shows that E18.5 ME cells exhibit substantially more molecular homology with basal than luminal cells, with little evidence of lineage bifurcation (Fig. 2a). The ternary plots also demonstrate that prepubertal cells (Fig. 2b) differ markedly from embryonic epithelial cells and are more similar to those in the adult gland (Fig. 2c). In 2-week-old glands (days 15–17), clusters corresponding to emergent basal, LP, ML could be detected, in addition to a luminal intermediate (Lum Int) cluster that was previously detected in pubertal and adult mammary glands upon scRNA-seq analysis [9] (Fig. 2b). These data indicate that lineage segregation commences prior to 2 weeks of age, in agreement with the findings of Giraddi et al. [6]. The scRNA-seq analysis of 4037 cells performed here differs from our previous analysis of 2-week-old glands (~ 120 cells), which resulted in the preferential isolation/capture of basal-like cells and led to the interpretation that segregation occurs closer to the onset of puberty [9].

To compare the molecular profiles of 2 week (prepubertal) epithelial cells with those in the early postnatal period, we performed scRNA-seq analysis on day 5 postnatal glands. The array of epithelial cell clusters at these two time points differed markedly (Fig. S2A, B). Based on expression of lineage marker genes, a basal (#4) and three basal-like clusters (#1, 3, 6) were distinguished at day 5, in addition to a diffuse luminal population (Fig. S2A, B), indicating the presence of basal- and luminal-biased cells shortly after birth. Both basal- and luminal-oriented intermediate cells were previously described at postnatal day 4 [6]; however, the basal compartment analyzed at postnatal day 5 shows substantially more heterogeneity with at least 4 clusters apparent (Fig. S2A, B). Cluster 4 in P5 glands may represent more mature myoepithelial cells based on expression of *Myh11* and *Acta2* (Fig. S2B), as well as *Lama1*, *Myl9* and *Mylk*. In 2-week-old prepubertal female mice, more defined lineages were evident (Fig. S2A, B), indicating evolution of the transcriptome during the period of isometric ductal growth between day 5 and days 15–17.

The compare cell populations more closely across the three developmental stages (embryonic, prepubertal, and adult cells from C57BL/6 mice), we integrated the three scRNA-seq stage-specific profiles and re-clustered, revealing 12 cell clusters and clear differences between the stages (Fig. 2d, e). The integrated profiles confirmed and complemented results from the individual profiles. The primordial epithelial cells from the E18.5 embryos were confirmed to harbor a unique transcriptional pattern that is basal-like but with lower basal signature expression than that of adult basal cells. The lineage identity of each cluster was indicated by the ternary plot and was further quantified by computing lineage expression signatures for each cell (Fig. 2f). As anticipated, the adult gland comprised basal, LP, ML, and Lum Int populations. While prepubertal cells showed overlap with adult basal cells, the two LP and ML populations in 2-week-old mice only partially overlapped with the definitive lineages in the adult, suggesting an intermediate state (Fig. 2e, ternary plot). The basal-like clusters 3, 6, and 10 contained mainly embryonic cells. Basal cluster 7 showed a wide range of basal signature expression and appeared to contain subclusters of embryonic, prepubertal, and adult cells with increasing levels of basal lineage gene expression. Exploration of potential lineage trajectories in pseudo-time further highlighted the unique nature of embryonic cells (Fig. 2g), comprising a small subset of basal-like intermediates, together with the bifurcation of postnatal cells into the definitive basal, LP and ML lineages.

Hierarchical analysis of the most highly expressed genes in each cell cluster across the different developmental stages highlighted the following features (Fig. S3A): (1) most cells in the embryonic rudiment exhibit a unique profile, showing little overlap with the luminal lineages; (2) the signature of embryonic basal-like cells differs from definitive basal/myoepithelial cells in the adult mammary gland. Embryonic mammary cells within cluster 7 are most basal-like but express low levels of many genes that characterize the adult basal compartment and high levels of *Igfbp2* and *Serpine2*; (3) embryonic clusters 3, 6, and 10 are enriched for genes associated with proliferating cells; (4) for prepubertal versus adult cells, differing profiles were observed for several ML-associated genes (e.g., *Krt7*, *Areg*, *Piezo2*, *Tspan13*, *Glu1*, *Epcam*, *Ets2*, *Jund*) as well as LP genes (*Epcam*, *Jund*, *Gas6*); and (5) clusters 11 and 12 exhibit mixed-lineage features (Fig. S3A). Expression plots for cardinal basal and luminal lineage-specific genes are shown in Fig. S3B. As anticipated, most but not all embryonic cells express the proliferation marker *Mki67*.

To gain insight into the molecular relationships between the clusters and the intrinsic breast cancer subtypes, expression scores for each of the 12 clusters were computed for 337 breast tumors of different subtypes [30] (Fig. S4). Embryonic mammary cluster 6 aligned with the basal-like subtype while clusters 7 and 10 were most concordant with claudin-low cancers. The largest embryonic cluster (#3) did not align with any particular subtype. As expected, the definitive cell lineages, basal, LP and ML, aligned most closely with the claudin-low, basal-like, and Lum A subtypes, respectively [31].

### Delineation of epithelial heterogeneity in dissected TEBs versus ducts at the single cell level

**Fig. 3:**
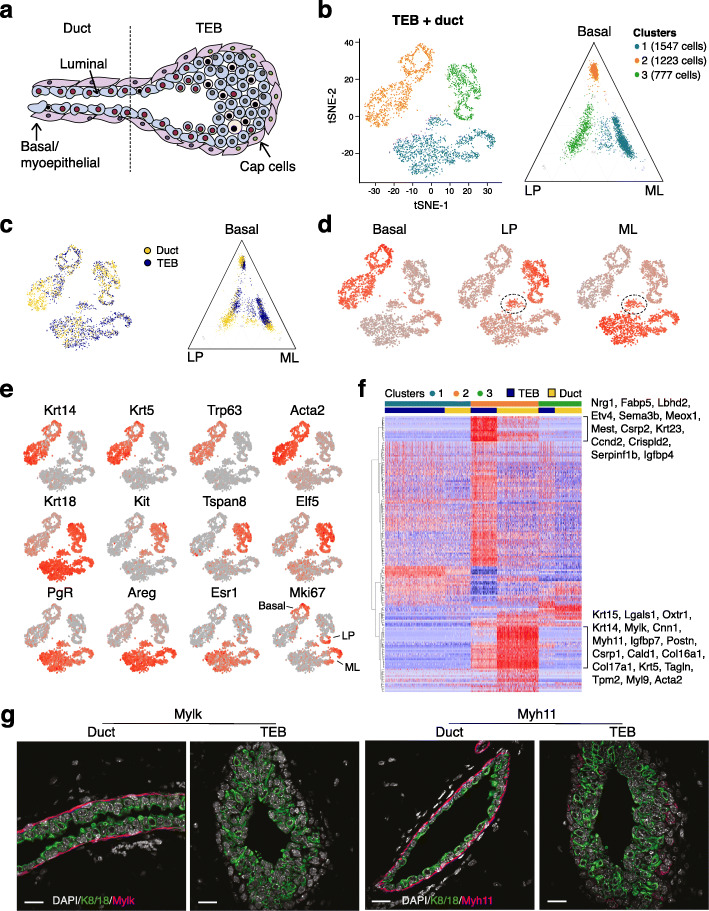
Single cell transcriptomes of terminal end buds and subtending mammary ducts in puberty. **a** Schematic diagram of the structure of the terminal end bud (TEB) that characterizes the ductal tree during puberty. **b** t-SNE and ternary plots of the integrated scRNA-seq transcriptomes of epithelial cells from microdissected TEBs and subtending ducts colored by cell cluster (n = 3 *Ecadherin-GFP* mice). Ternary plot positions the cells by expression of known lineage signature genes as described above. **c** Same t-SNE and ternary plots as in **b** but colored by cellular structure: TEBs (blue) and ducts (yellow). **d** Same t-SNE plot as in **b** and **c** but colored by basal, LP and ML signature scores. The cells marked with a dotted circle express both LP and ML signatures and match the intermediate LP-ML subset visible in the ternary plot in **b**. **e** t-SNE expression plots for typical epithelial markers and the proliferation marker Mki67. **f** Heatmap showing relative expression for 164 genes DE between TEB (blue) vs ductal (yellow) cells in the basal compartment. Red = high expression; blue = low expression. **g** Co-immunofluorescence labeling of sections from 5-week-old glands (TEBs and ducts) for expression of Keratin K8/K18 (green), Mylk, or Myh11 (pink). DAPI is shown in gray. Scale bars, 20 μm

To further understand molecular heterogeneity within the TEB structures that drive ductal morphogenesis [3], mammary ducts and TEB regions were dissected from 5-week-old *E-cadherin-GFP* knock-in mice [15] using GFP to highlight TEBs under a stereomicroscope (Fig. 3a and Fig. S5A). Epithelial cells (Lin^–^CD24^+^CD29^+^) were sorted from the micro-dissected portions by flow cytometry and then captured for scRNA-seq analysis (Fig. S5B-D). The ductal and TEB single cell profiles were integrated and cells were clustered into three dominant populations (Fig. 3b, c). Positioning cells on a ternary plot identified the three clusters as corresponding to ML, basal, and LP populations respectively and positioned a subset of cluster 1 as a LP-ML intermediate population (Fig. 3b). These cluster identities were confirmed by coloring cells by their expression of basal, LP, and ML signature scores (Fig. 3d). The luminal populations (both LP and ML) in the body layers of TEB structures appear to harbor a more intermediate signature relative to ductal luminal cells (Fig. 3c). The expression of canonical luminal and basal-specific genes plotted on the different clusters indicated that each major population comprised a substantial subset of proliferating *Mki67+* cells (Fig. 3e). Consistent with recent findings [32], *Tspan8* was not expressed in basal cells of the TEB units.

Differentially expressed (DE) genes were identified between TEB and ductal cells within each of the three clusters in Fig. 3b, resulting in 164, 28, and 35 DE genes for the basal, LP, and ML clusters respectively (Bonferroni adjusted p < 0.05). Figure 3f shows expression values by cell for the 164 basal DE genes. Genes preferentially expressed in TEBs include *Nrg1*, *Sema3b*, *Meox1*, and *Ccnd2* while *Postn*, *Col16a*, and genes important for contractile activity (*Myh11*, *Myl9*, *Mylk*, *Acta2*), were among the top DE genes for duct-enriched basal cells. Co-immunofluorescence staining for Myh11 (heavy chain) or Mylk (light chain) confirmed substantially higher levels in the basal layer of ducts (Fig. 3g). Thus, genes required for myoepithelial cell contractility and extracellular matrix proteins were found to be significantly upregulated in ductal versus TEB basal cells of pubertal mammary glands.

### Differential chromatin accessibility in basal cells of TEB and ductal structures

**Fig. 4:**
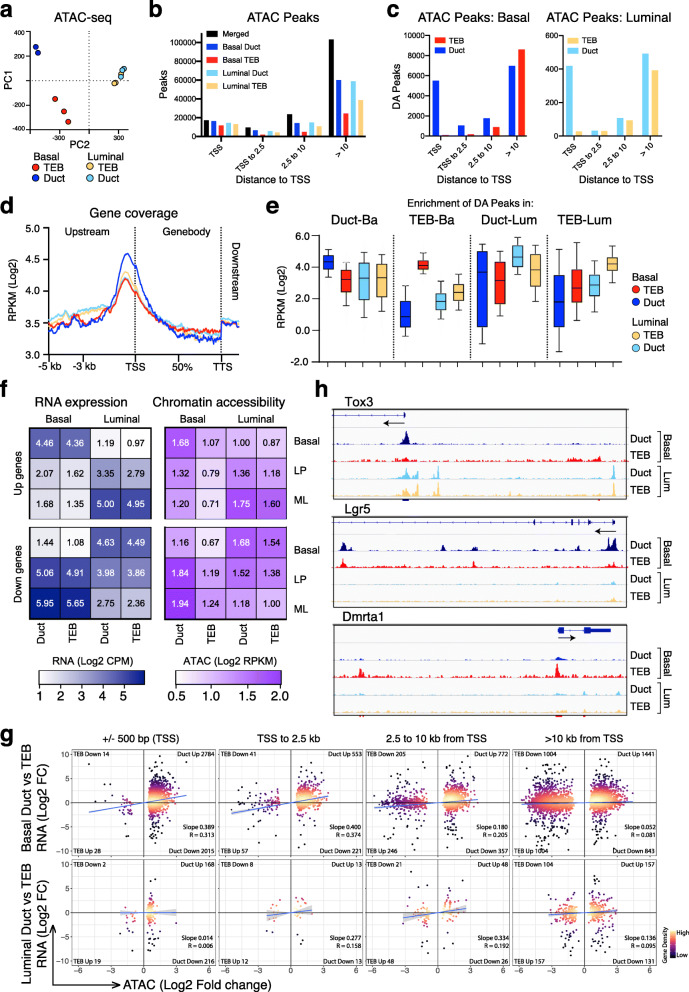
Bipotent promoter chromatin accessibility is established within basal ductal cells during puberty. **a** Principal component analysis (PCA) of ATAC-seq profiles (represented by log2-RPKM intensities for merged peaks) for TEB and duct basal and luminal populations. **b** Number of peaks identified in each cell population classified by distance to the nearest transcription start site (TSS). Overlapping peaks between cell populations have been merged to make consistent peak boundaries across all populations. Black bars show total number of peaks after merging those called in each of the four cell populations (colored bars). **c** Differential accessibility between TEBs and ducts for the basal and luminal populations respectively (FDR < 0.05). Peak genomic regions are categorized based on the distance to their nearest TSS as in **b**. **d** Chromatin accessibility averaged over all genes from 5 kb upstream of TSS to 1 kb downstream of transcription termination site (TTS). **e** Average intensities of DA peaks in the different cell populations. Panels from left to right show peaks that are significantly more accessible in ductal vs TEB basal cells, TEB vs ductal basal cells, ductal vs TEB luminal cells, and TEB vs ductal luminal cells respectively. Within each panel, boxes show average log2-RPKM of the same peak regions in basal ducts (blue), basal TEBs (red), luminal TEBs (yellow), and luminal ducts (light blue). Boxes show median, quartiles and 10th and 90th percentiles. **f** Average RNA expression and chromatin accessibility of lineage signature genes. Top panels show results for positive signature genes for the basal, LP, and ML epithelial cell populations respectively and lower panels show results for negative signature genes [19]. Left panels show average log-expression (log2 counts per million) from bulk RNA-seq for the signature genes in basal and luminal cells from ducts and TEBs. Right panel shows average ATAC-seq coverage within 10 kb of the TSS of the same genes in the same cell populations. ATAC-seq coverage is represented as log2(RPKM+1) for the 20 kb TSS neighborhoods, ignoring ATAC-seq reads not associated with a MACS peak. **g** Scatterplots of genewise expression changes in TEBs vs ducts vs accessibility changes in the same genes. Expression and accessibility are both represented as log2-fold-changes (log2 FC). Upper panels show results for basal cells, lower panels for luminal cells. Panels left to right show results for ATAC-seq peaks within 500 bp of a TSS, 0.5–2.5 kb from a TSS, 2.5–10 kb from a TSS or > 10 kb from a TSS. Only peaks that are DA for the relevant comparison are plotted. Each DA peak is matched with the expression fold-change for the nearest gene. For each plot, the number of genes in each quadrant is shown, along with the linear regression slope and R value. **h** Coverage plots of chromatin accessibility in the different cell populations for key lineage-specific genes

To better understand the molecular features of TEBs, we further analyzed the cellular compartments of TEBs and ducts by Assay for Transposase Accessible Chromatin (ATAC) sequencing. Given the limiting numbers of cells from dissected structures, a bulk population approach was adopted. Principal component analysis (PCA) revealed that chromatin accessibility differed dramatically between TEB and duct cells for the basal compartment (Fig. 4a). Peak analysis of basal and luminal populations for TEBs and ducts demonstrated that sites distal to the transcriptional start site (TSS) tend to exhibit more variable accessibility between cell types, and result in a large number of unique peaks, especially beyond 10 kb from the nearest TSS, when merged (black bars) (Fig. 4b). However, peaks were more frequent per kb close to the TSS rather than at distal sites. While thousands of sites demonstrated differential accessibility in the basal populations (Fig. 4c), a smaller number of loci differed between the TEB and duct luminal populations. Relative to duct cells, differentially accessible (DA) peaks in the TEB populations were predominantly found distal to the TSS. Interrogation of genome-wide accessibility of genes showed a high level of accessible chromatin that was proximal to or overlapping the TSS within the basal duct versus the basal TEB population (Fig. 4c, d). Moreover, many peaks enriched in the ductal luminal populations were observed to be highly accessible in basal duct cells (Fig. 4e). By contrast, most DA peaks in basal duct cells were less intense in the luminal duct population. The chromatin accessibility of lineage signature genes (TSS and the surrounding ± 10 kb) correlated with their expected expression in the cell populations (Fig. 4f). Despite ductal basal cells exhibiting strong expression of the Basal-up, LP-down, and ML-down signatures, their chromatin appeared open at the TSSs of most signature genes (Fig. S6A). This bi-lineage chromatin profile was less apparent in TEB-enriched basal cells, which showed a significant decrease in accessibility for all signature gene comparisons (e.g., P values of 2.58 × 10^−8^, 2.08 × 10^−8^, and 1.45 × 10^−8^ for LP-up, ML-up, and Basal-down signatures, respectively) (Fig. 4f and Fig. S6A). These data suggest that bi-lineage chromatin profiles are established following ductal elongation. Compatible with this finding, most DA peaks within the TEB basal population were highly accessible only in these cells, contrasting with DA peaks in the TEB luminal population, which appeared more accessible in other cell types (Fig. 4e). Further investigation of TEB basal peaks revealed that 13% resided within repeat elements (Fig. S6B). These may be involved in defining the basal chromatin landscape within TEBs but this is yet to be determined.

We next correlated differential gene expression with accessibility changes in the vicinity of genes. As expected, increased accessibility was positively correlated with increased expression, especially within 2.5 kb of a TSS (Fig. 4g and Fig. S6D). Accessibility of peaks within the basal duct population strongly correlated with gene expression, as 60–70% of basal duct DA peaks demonstrated increased accessible chromatin and expression of their nearest gene regardless of distance to the TSS (Fig. 4g and Fig. S6D). In the other populations, this association was essentially limited to the TSS and proximal region. The association of distal element (beyond 2.5 kb from a TSS) accessibility with gene expression appeared stronger across cell types than between cellular structures (ducts vs TEBs). DA peak analysis of basal versus luminal populations (Fig. S6C) showed that > 66% of peaks greater than 2.5 kb from a TSS that are more accessible in basal cells positively associate with gene expression (Fig. S6D), suggesting that distal elements (putative enhancers) within the basal population affect their nearest gene.

Since duct basal cells exhibited a high level of TSS accessibility genome-wide, whereas the luminal subsets tended to show increased accessible chromatin within a 10 kb region proximal to the TSS (Fig. 4c and Fig. S6C), we further analyzed the regions surrounding lineage-specific signature genes (Fig. S6A). For genes within the Basal-up, LP-down, and ML-down signatures, each basal population exhibited a high level of accessible chromatin at the TSS and within the surrounding 10 kb, reflecting a strong correlation between accessibility and gene expression. The luminal and basal ductal populations exhibited similar TSS accessibility for signature genes. In luminal cells, the genes in the ML-up and Basal-down signatures displayed an increased level of chromatin accessibility proximal to the TSS (± 10 kb), which was not seen in basal cells. Examples of TSS proximal-acquired accessibility in luminal cells can be seen for luminal genes including the transcription factor *Tox3* and the fatty acid gene *Faah* (Fig. 4 h and Fig. S6E). Interestingly, accessibility of peaks within 10 kb and peaks > 10 kb from genes in the LP- and ML-down and Basal-up signatures was enriched in luminal cells. These open sites may represent novel silencer elements.

The TSS was found to be highly accessible in canonical basal genes including *Krt5* and *Postn* for basal duct and TEB cells and *Lgr5* in ductal cells (Fig. 4h and Fig. S6E). Of note, expression of *Lgr5* has been previously shown to be restricted to basal duct cells [33, 34]. Very few gene TSSs, such as *Dmrta1* and *Htr5b*, exhibited TEB-specific accessible chromatin together with enrichment of expression (Fig. 4 h and Fig. S6E). No difference in expression or accessibility was found between the TEB versus duct regions for the luminal marker genes *Krt8* and *Krt18* (Fig. S6E). Taken together, these data corroborate the role of chromatin accessibility in defining lineage-specific gene expression. The genome-wide increase in promoter accessibility in ductal basal cells may be linked to the renewal, activation and/or differentiation potential of these cells.

### Comparison of scRNA-seq expression profiles of different adult strains

**Fig. 5:**
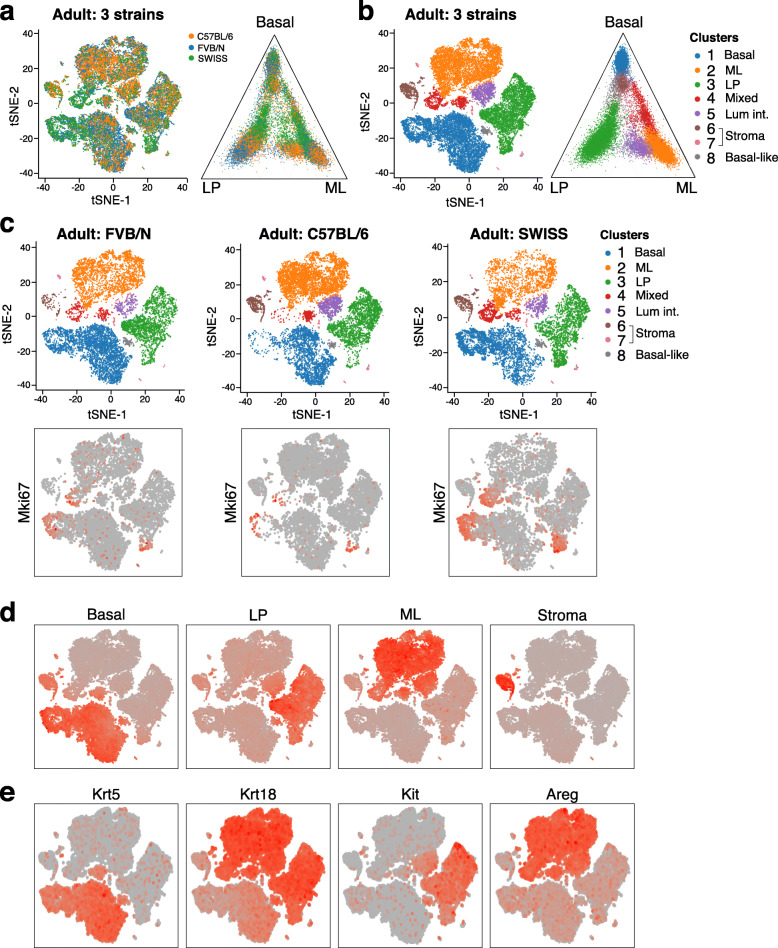
Comparison of single cell transcriptomes of different strains. **a** t-SNE and ternary plots of the combined scRNA-seq transcriptomes of epithelial cells isolated from adult mammary glands (10 weeks) from three different mouse strains: FVB/N, C57BL/6, and SWISS (SW) (n = 3–4 pooled mice per strain), colored by strain. Mice were not staged for the estrus cycle. Mixed refers to mixed-lineage. **b** Same plots as **a** but colored by cell cluster. **c** Upper panels, same integrated t-SNE plot as in **b** but shown separately for each individual strain. Lower panels color cells by *Mki67* expression instead of cluster. **d** Same t-SNE plot as **a** but colored by basal, LP and ML expression signatures. Red = high expression, gray = no expression. **e** Same t-SNE plot as **a** but colored by expression of lineage-specific marker genes

As different mouse strains have been used in the field for scRNA-seq studies, we investigated the level of similarity across them by determining the single cell transcriptomes of adult mammary epithelial cells derived from three different strains: C57BL/6, FVB/N and SWISS (CD1-like). All strains appeared similar by scRNA-seq analysis (Fig. 5a). Clustering identified the usual basal, LP, and ML populations and some intermediates (Fig. 5b). Separating the t-SNE plot by strain indicated that C57BL/6 glands have less cells in a subcluster of the basal compartment, while SWISS glands were noted to contain more *Mki67*+ cells within each of the major subsets (basal, LP, ML) (Fig. 5c). These proliferative clusters may reflect different stages of the estrus cycle [8, 9] or higher numbers of proliferative intermediates in SWISS mice. Overlaying the basal, LP, ML, and stromal expression signatures onto the combined t-SNE plot showed expression of the appropriate signature in each of the three major epithelial clusters and identified a small stromal population (Fig. 5d), with lineage markers shown in Fig. 5e. In summary, although molecular differences between the strains were detectable, further analyses are required to determine whether SWISS mice comprise more cellular intermediates.

### A continuum of luminal progenitor-like intermediates revealed through an integrative analysis of transcriptomes through the pregnancy cycle

Previous analysis of the pregnancy-lactation-involution cycle at the single cell level by Bach et al. yielded 15 potential cell clusters [5]. Here, we have expanded on the pregnancy cycle through the inclusion of three additional time points (spanning pregnancy and post-involution) in order to further define changes in the epithelium occurring across the reproductive cycle. Lin^–^CD24^+^CD29^hi^ mammary epithelial cells were sorted from FVB/N mice at the following stages: adult, mid-pregnancy (day 12.5), late pregnancy (day 18.5), lactation (2 weeks), and post-involution (3 weeks post-weaning), when the gland has undergone remodeling.

**Fig. 6:**
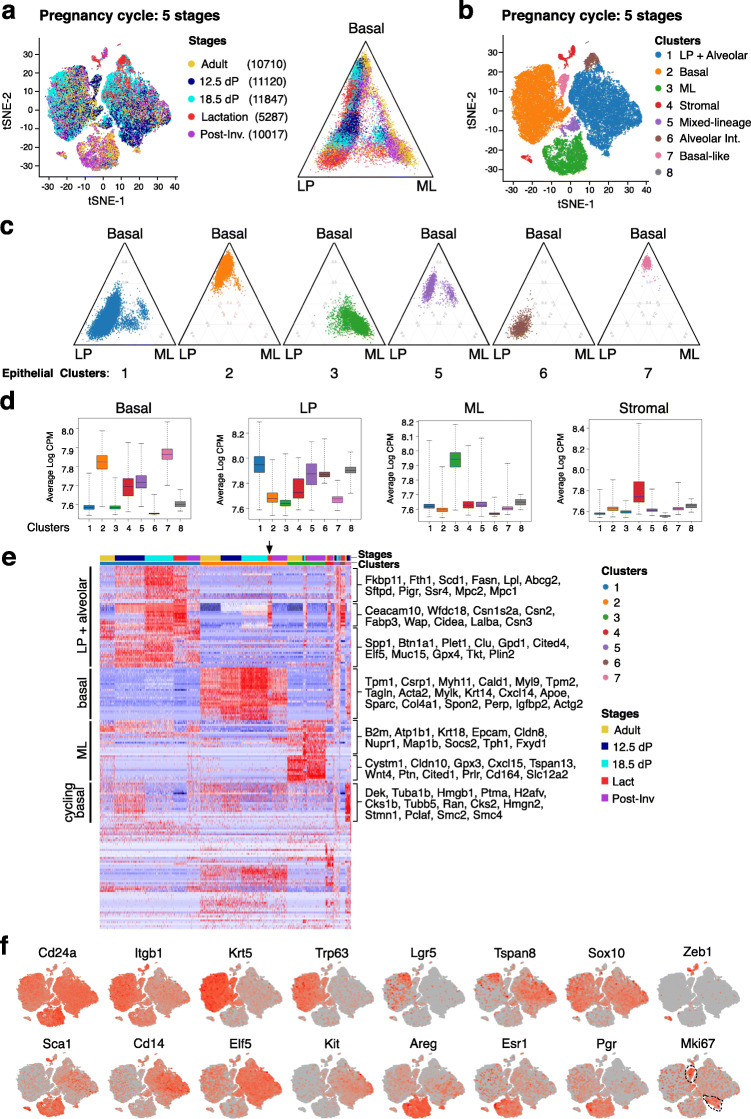
Integration of single cell transcriptomes across the pregnancy cycle reveals intermediate states. **a** t-SNE and ternary plots of the integrated scRNA-seq profiles of epithelial cells isolated from adult (10 weeks), mid-pregnant (12.5 dP), late-pregnant (18.5 dP), lactating (8 day), and post-involution (3 weeks post-weaning) mammary glands from FVB/N mice (n = 4 mice for adult; n = 2 pooled mice for other stages). Cells are colored according to stage. The larger representation of alveolar cells in pregnancy and lactation reflects their abundance. **b** Same t-SNE plot as in **a** but colored by cell clusters. **c** Individual ternary plots showing lineage identity for each cell cluster from **a**. **d** Boxplots of signature expression scores for each of the 8 clusters in **b** by mammary epithelial or stromal subtype. **e** Heat map showing relative expression of the top 30 marker genes for each cluster in **b**. Color bars at the top of the heatmap represent the cluster and the stage. The arrow depicts “lineage-primed” cells in the basal compartment of lactating glands. **f** t-SNE plots (as in **b**) showing the expression of markers used for cell fractionation and definitive lineage markers and *Mki67*

Analysis of biological replicate samples for the adult, mid-pregnancy, late-pregnancy, and lactation stages showed strongly reproducible cell subsets between replicates for each developmental stage (Fig. S7A-D), so the comparative analysis between stages was conducted using a single representative sample at each stage. Single cell transcriptomes were obtained for 48,981 epithelial cells (adult, A10; 12.5 dP (days pregnancy), H; 18.5 dP, F4; lactation, F11; post-involution) after quality control and filtering (Table 1). Integration of the single cell profiles across the five stages resulted in 8 clusters, including the three anticipated major populations (clusters 1–3) and four small clusters (clusters 4 to 7) (Fig. 6a, b), with the contribution of cells from different stages of morphogenesis shown in Fig. 6a. Notably, the ternary plot suggests a continuum of cellular states, particularly along the Basal-Luminal Progenitor axis.

The lineage identity of each cluster was determined by ternary plot analyses (Fig. 6c; Fig. S7E) and quantified by computing lineage expression signatures for each cell (Fig. 6d). The latter analysis revealed novel epithelial intermediates and identified cluster 4 as stromal cells. Interestingly, cluster 6 appears to comprise a distinct subset of alveolar intermediates that emerge in late pregnancy and are enriched during lactation. These cells are distinguished by high levels of milk protein genes (e.g., *Wap*, *Csn1-3*, *Lalba*, *Wfdc18*) and lower levels of LP genes (such as *Elf5*) and several alveolar genes characteristic of cluster 1 in lactation (Fig. 6e). Thus, cluster 6 may represent a cellular intermediate that lies between LP cells and the differentiated alveolar cells that predominate in lactating glands. This cluster seems to differ from C10 identified in 14.5-day pregnant glands [5] as it was not detectable at day 12.5 pregnancy (Fig. 6e). The basal compartment represented by cluster 3 comprised a subset of *Lgr5*^*+*^ and *Tspan8*^*+*^ cells, previously reported to mark quiescent mammary stem cells [33]. The distribution of expression of these genes within the different clusters as well as expression of other canonical markers is shown in Fig. 6 f. Interestingly, a subset of “alveolar lineage-primed” cells expressing milk protein transcripts was evident within the basal compartment during lactation (Fig. 6e). Cells within the smaller basal cluster 7 expressed a myriad of cell cycle genes (e.g., *Mki67*, *Tuba1b*, *Hmgb1*, *H2afv*, *Cks2*, *Pclaf)*; these proliferating basal progenitor cells were most prevalent in mid-pregnancy (Fig. 6e, f) but were also detected in the adult gland where they likely represent cycling basal cells in diestrus [9]. A cycling basal cluster is evident in the replicate plots for 12.5 days pregnancy (Fig. S7B), while a distinct cluster (#4) of differentiated myoepithelial cells in lactating glands (expressing abundant contractile genes including *Mylk*, *Myh11*, *Myl6*, *Myl9*, *Mme*, *Acta2*) is most clearly visualized in Fig. S7D (and data not shown).

In contrast to the uni-lineage clusters, cluster 5 comprised two subsets, each displaying mixed-lineage features (co-expression of canonical basal and luminal genes) (Fig. 6c). This cell cluster was expanded in pregnancy and comprised 2.5% of the overall population. Collectively, these data demonstrate that both the luminal and basal compartments change their molecular properties according to developmental stage and that a number of alveolar progenitor states can be discerned along the basal-LP axis, indicating a dynamic shift occurs within the mammary epithelium concomitant with alveologenesis.

## Discussion

**Fig. 7:**
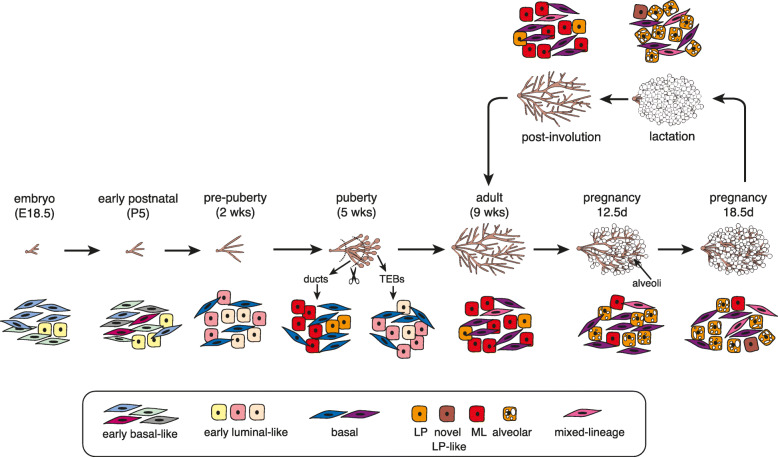
Schematic diagram summarizing epithelial heterogeneity unveiled in the mouse mammary gland at 9 different stages of development by scRNA-seq analysis: E18.5, postnatal day 5, prepuberty (2-week), puberty, adult, mid-pregnancy, late-pregnancy, lactation, and post-involution. Heterogeneity in the post-involution and virgin mammary glands appeared similar

This large-scale atlas builds on other scRNA-seq reports [5–10] through the sequencing of large numbers of single cells at additional stages (prepuberty, two further time points in pregnancy, post-involution), as well as dissected TEBs and subtending ducts, and two closely associated embryonic ectodermal lineages (skin and the mammary primordia). Our previous scRNA-seq analysis using the 10x Genomics Chromium platform focused on cells from puberty, adulthood, and the estrus cycle [9]. A schematic representation of epithelial heterogeneity uncovered across the main developmental stages reported here is shown in Fig. 7.

The precise timing of specification of the definitive lineages in the mammary gland remains unclear. Lineage tracing experiments with specific gene promoters have suggested that cells are poised for lineage restriction prior to birth and that this may occur as early as E15.5 [35–37]; however, distinct subsets of cells could not be identified based on their transcriptomes. In this study, interrogation of the embryonic population indicated that embryonic cells form a broad population that is unique but molecularly closer to the basal lineage. No hybrid-lineage signatures or evidence for lineage segregation was observed, mirroring that described for Epcam^+^ fetal cells at E18.5 [6]. Single cell profiling and diffusion analysis of postnatal day 4 cells has suggested that they occupy an intermediate position between the embryonic and adult mammary populations [6]. Single cell analysis of day 5 postnatal cells here indicated a heterogeneous population of basal-like cells and a luminal cluster, whereas 2-week-old mice exhibited lineage commitment towards the definitive basal, LP and ML lineages. However, neither LP or ML cells from prepubertal mice expressed the gene signatures that characterize the adult populations. Although cell clustering of 5 week-old pubertal glands resembles that seen in the adult gland by scRNA-seq analysis [9], finer dissection of TEB versus ductal structures revealed that TEBs harbor more intermediate single cell expression profiles. Overall, these data suggest that while lineage commitment is initiated in the early postnatal period, the definitive lineage-specific expression programs are completed during ductal morphogenesis in puberty.

Both the basal and luminal compartments appear to be dynamically regulated through the pregnancy-lactation cycle. Through an integrated transcriptomic analysis of almost 49,000 single cells, we show that the basal-LP axis is particularly fluid across the different developmental stages, with a number of intermediates lying between these two populations, potentially forming a continuum of states. A novel cluster of “alveolar” intermediate cells (cluster 6) was induced in late-pregnancy and persisted in lactation but was not present in the adult or mid-pregnant gland. In addition, the mixed-lineage cluster (cluster 5) was most prominent during pregnancy. These cells may represent a transient population of lineage-primed cells that is poised for commitment to the luminal lineage. Interestingly, functional analysis of a c-Kit+ basal subpopulation, reminiscent of this lineage-primed subpopulation, revealed that they act as facultative mammary stem cells [38]. In the context of normal human breast tissue, single cell profiling and pseudo-temporal differentiation trajectories produced a continuous lineage linking the basal and two luminal arms [10], while another study suggested that the stem-like state in human breast is associated with bipotency and clinical outcome [39]. In other systems, the identification of lineage-primed or multipotent cells through single-cell analysis of hematopoietic, pancreatic, and intestinal cells has provided important insights into rare cellular states [40–43].

An in-depth analysis of micro-dissected TEBs and their subtending ducts from pubertal mice undergoing ductal morphogenesis indicated that the two primary lineages share similar clustering at the single cell level. Luminal cells (both LP and ML) within enriched TEBs appeared to harbor less mature signatures, consistent with the notion that TEBs predominantly comprise progenitor-like cells. Moreover, basal cells within TEBs were less differentiated than their ductal counterparts and expressed dramatically lower levels of extracellular matrix proteins and myosin filament proteins required for the contractility of myoepithelial cells. Bulk RNA-seq of TEB versus ductal-derived cells confirmed the less differentiated state of basal cells within TEBs (data not shown). By contrast, previous scRNA-seq of a small number of pubertal cells showed two main clusters but suggested that progenitor cells within TEBs do not have a specific gene signature [44].

Investigation of the transcriptional and chromatin accessibility landscapes of TEBs and subtending ductal regions in pubertal glands revealed that TEBs exhibit reduced chromatin accessibility compared to cells within mature ducts. Interestingly, the TSSs of luminal signature genes were highly accessible in basal duct cells relative to TEB basal cells, implying that lineage-specific chromatin is established in the subtending ducts. Although less enriched in bi-lineage chromatin, basal TEB cells demonstrate a distinct chromatin landscape, where many accessible sites were highly enriched in these cells and mapped to repeat regions. Although there was a significant correlation between gene expression and TSS chromatin accessibility within each population, changes in accessibility more closely reflected transcriptome changes in the basal rather than the luminal populations. The TSS region was the strongest indicator of gene expression changes between basal and luminal populations (either TEB or duct) but many distal elements in basal cells strongly correlated with expression of the nearest gene. Interestingly, the acquisition of regulatory elements proximal to the TSS (within 10 kb) in luminal cells strongly correlated with gene expression but additional sites more than 10 kb from the TSS negatively associated with expression. These negatively associated distal elements may represent novel silencer sites, which are proving to be critical for development [45, 46]. Reminiscent of our findings for basal cells in puberty, adult mammary basal cells were shown to have more open chromatin than luminal cells (LP and ML), with most of the increased chromatin accessibility occurring in distal regions [11]. Moreover, embryonic mammary stem-like cells and basal adult cells both exhibit chromatin accessibility and epigenetic features indicative of multilineage differentiation potential [11]. Recent single cell ATAC-seq analysis showed the presence of E18.5 mammary cells with either luminal or basal-oriented chromatin features [12]. Given the lag that occurs between chromatin changes and transcriptional expression, these data suggest that embryonic cells exist in a unique state and are poised for lineage restriction in the postnatal period.

Exploration of molecular heterogeneity among individual mammary cells at a global level has revealed different levels of diversity within the luminal and basal compartments during mammary ontogeny that were not possible to uncover using bulk RNA-seq methodology. Recent single cell data also suggest that both epithelial compartments together with the surrounding stroma change upon aging [47]. Although there are some differences in the epithelial clustering apparent between different studies, this likely reflects the cell preparation, depth of sequencing, and the precise algorithms used for bioinformatic analyses. It is important to note that transcript expression can be transient and that other molecular features such as chromatin accessibility and epigenetic modifications need to be considered for an in-depth molecular understanding of regulatory states.

## Conclusions

This large-scale integrative analysis of > 132,000 individual cell transcriptomes of mammary epithelial cells spanning late embryogenesis to post-involution highlights the impact of developmental phase on molecular and cellular heterogeneity. The study revealed that mammary embryonic cells are far closer to the basal than luminal lineage and unveiled intermediate populations in prepubertal glands, TEBs in puberty, and alveoli in pregnancy. During progression of the pregnancy-lactation cycle, dynamic luminal progenitor subsets were identified along the basal-LP axis. Interestingly, lineage-specific chromatin seems to be laid down in the ductal (and not TEB) cells during puberty. Collectively, this atlas provides a valuable resource for understanding how heterogeneity evolves in the developing mammary gland.

## Supplementary Information

**Additional file 1:** **Figure S1.** Cell sorting of E18.5 mammary gland (MG) and associated skin cells. **Figure S2.** Molecular heterogeneity in mammary epithelial cells from four stages spanning embryogenesis to adulthood. **Figure S3.** Molecular heterogeneity in mammary epithelial cells spanning embryogenesis, prepuberty and adulthood. **Figure S4.** Relationship between cancer subtypes and normal epithelial subtypes during development. **Figure S5.** Cellular analysis of microdissected TEBs and ducts. **Figure S6.** Differential accessible peaks identified from ATAC-sequencing analysis of dissected TEBs and ducts. **Figure S7.** Biological replicates show consistent cell subsets at each developmental stage. **Table S1.** scRNA-seq quality control statistics.

## Data Availability

All data generated and analyzed during this study are included in this published article and its supplementary information files and have been deposited in the GEO database (GSE164017 and GSE164307).
