# Supplementary material for: Single cell transcriptome atlas of mouse mammary epithelial cells across development
Source: Breast Cancer Res. 2021 Jun 29;23:69. doi: 10.1186/s13058-021-01445-4 (PMC8243869; doi:10.1186/s13058-021-01445-4)
Supplement: Supplementary file 1 — Additional file 1: Figure S1. Cell sorting of E18.5 mammary gland (MG) and associated skin cells. Figure S2. Molecular heterogeneity in mammary epithelial cells from four stages spanning embryogenesis to adulthood. Figure S3. Molecular heterogeneity in mammary epithelial cells spanning embryogenesis, prepuberty and adulthood. Figure S4. Relationship between cancer subtypes and normal epithelial subtypes during development. Figure S5. Cellular analysis of microdissected TEBs and ducts. Figure S6. Differential accessible peaks identified from ATAC-sequencing analysis of dissected TEBs and ducts. Figure S7. Biological replicates show consistent cell subsets at each developmental stage. Table S1. scRNA-seq quality control statistics. [file 13058_2021_1445_MOESM1_ESM.pdf]

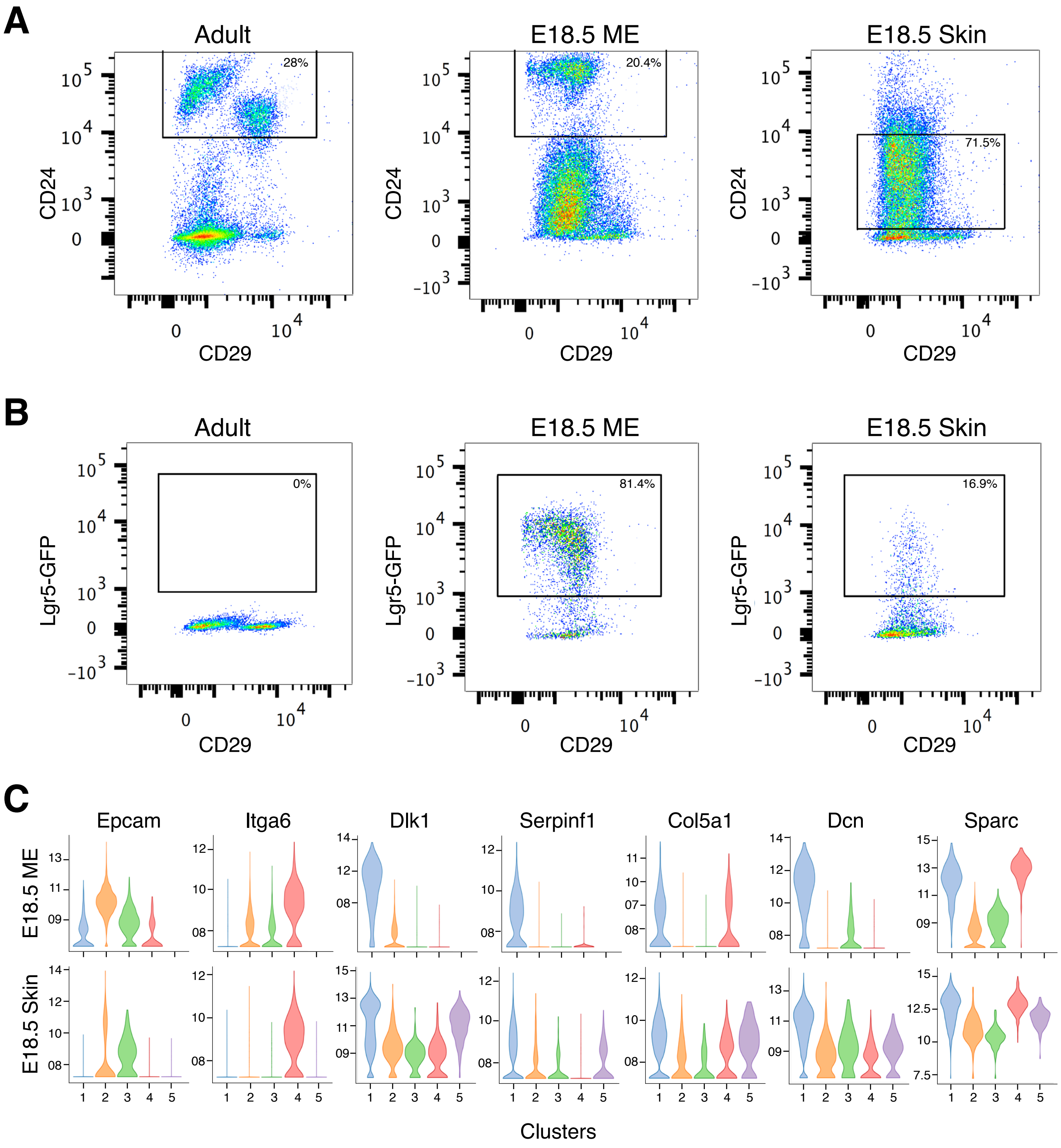

Figure S1. Cell sorting of E18.5 mammary gland epithelium (ME) and associated skin cells. (**A, B**) FACS analysis of adult mammary glands, embryonic mammary glands and skin (n=7 female embryos). Lineage-negative cells were sorted based on CD24 expression using the adult mammary gland to set gates. The ME and skin cells collected at E18.5 are indicated. FACS plots based on gating for $CD24^{+}$ cells are shown in (A), and their expression of CD29 and *Lgr5*-GFP shown in (B). (**C**) Violin plots showing expression of selected epithelial, epidermal and fibroblast genes in each cell cluster for either E18.5 ME or adjacent skin cells. Vertical axis shows expression as log2-CPM.

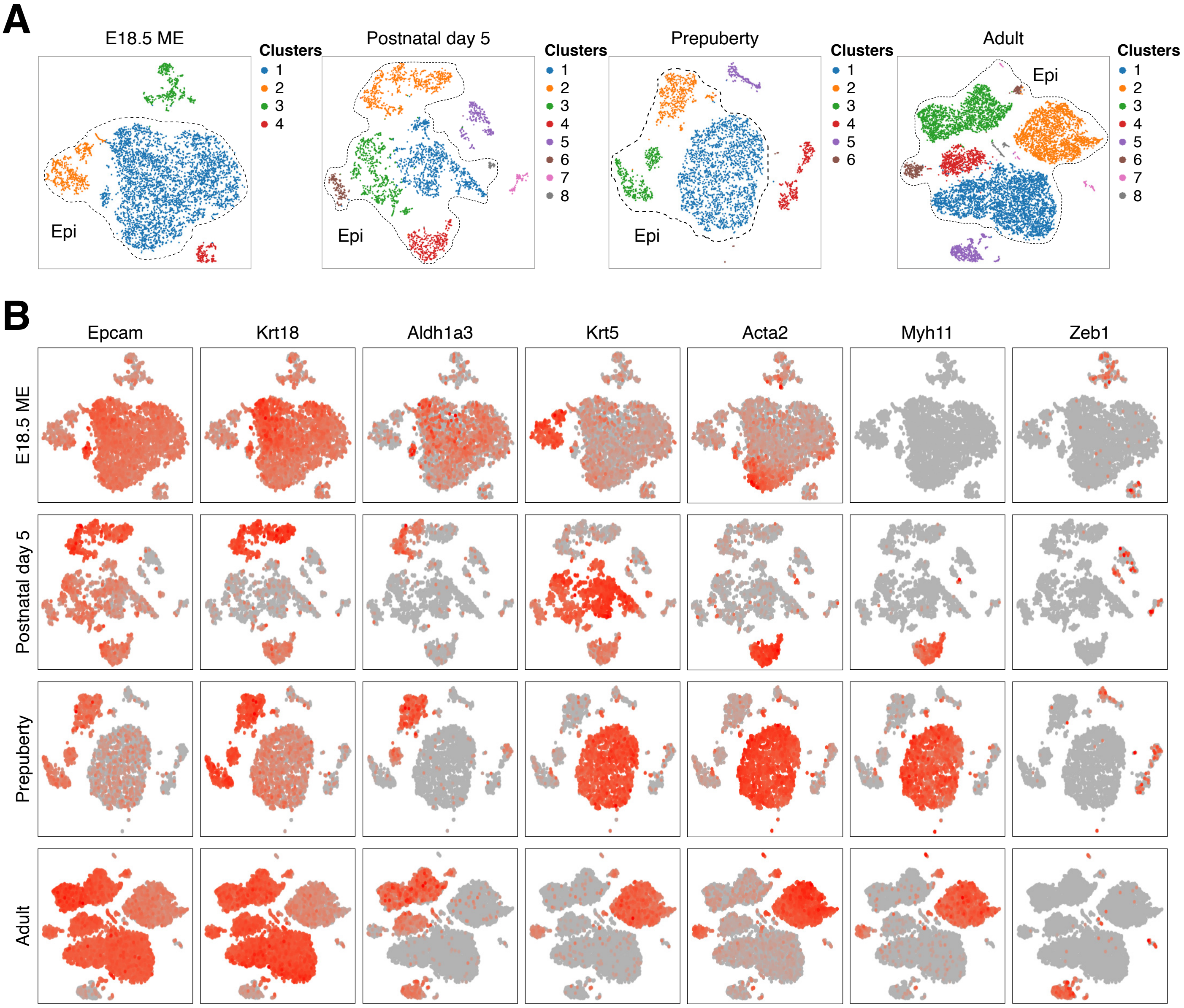

Figure S2. Molecular heterogeneity in mammary epithelial cells from four stages spanning embryogenesis to adulthood. (**A**) t-SNE plots showing clustering for single cell transcriptomes of mammary epithelial cells from embryonic (E18.5), early postnatal (day 5), prepubertal (2 weeks) and adult (10 weeks) mice. Cells were obtained from n=7 C57BL/6 embryos, n=35 C57BL/6 mice for day 5, n=35 C57BL/6 mice for 2 weeks and n=4 C57BL/6 adult mice. Clusters inside the dotted lines were considered to contain epithelial cells. Clusters outside the marked region were considered to comprise contaminating skin or stromal cells. (**B**) Same t-SNE plots as (A) but colored by expression of epithelial lineage (luminal, *Krt18*; basal, *Krt5*, *Acta2*, *Myh11*; LP, *Aldh1a3*) and stromal marker (*Zeb1*) genes.

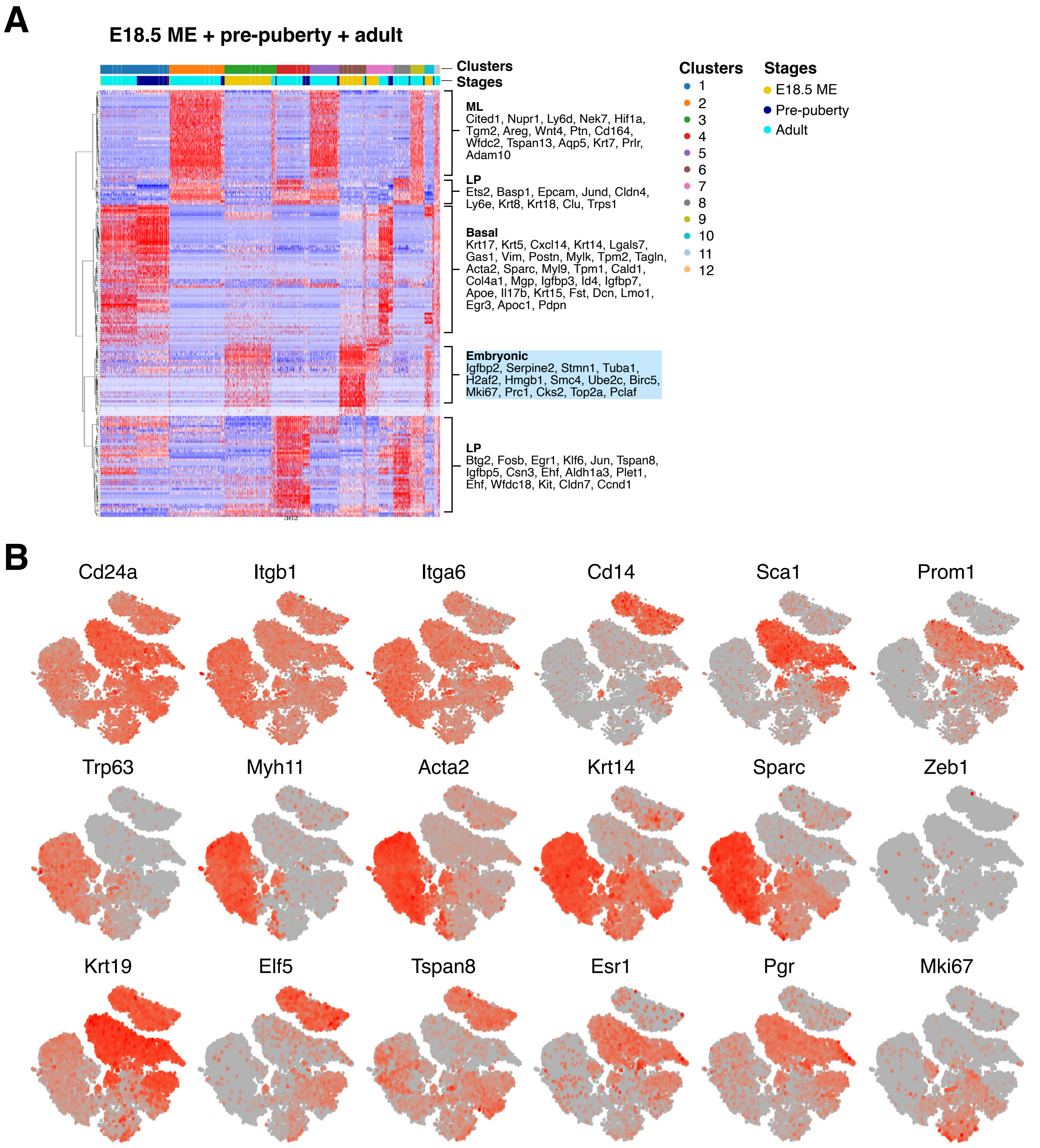

Figure S3. Molecular heterogeneity in epithelial cells spanning embryogenesis, prepuberty and adulthood. (**A**) Heat map showing relative expression of the top 30 marker genes for each of the clusters identified in Fig. 2d. The blue box indicates embryonic and cell cycle genes. (**B**) t-SNE plots showing expression of key markers of basal (*Trp63, Acta2, Krt14, Sparc*), luminal progenitor (*Elf5, Tspan8*), mature luminal (*Esr1, Pgr*) cells and a proliferative gene (*Mki67*). Markers commonly used for fractionation are also shown (*Cd24a, Itgb1, Itga6, Sca1, Cd14, Prom1*).

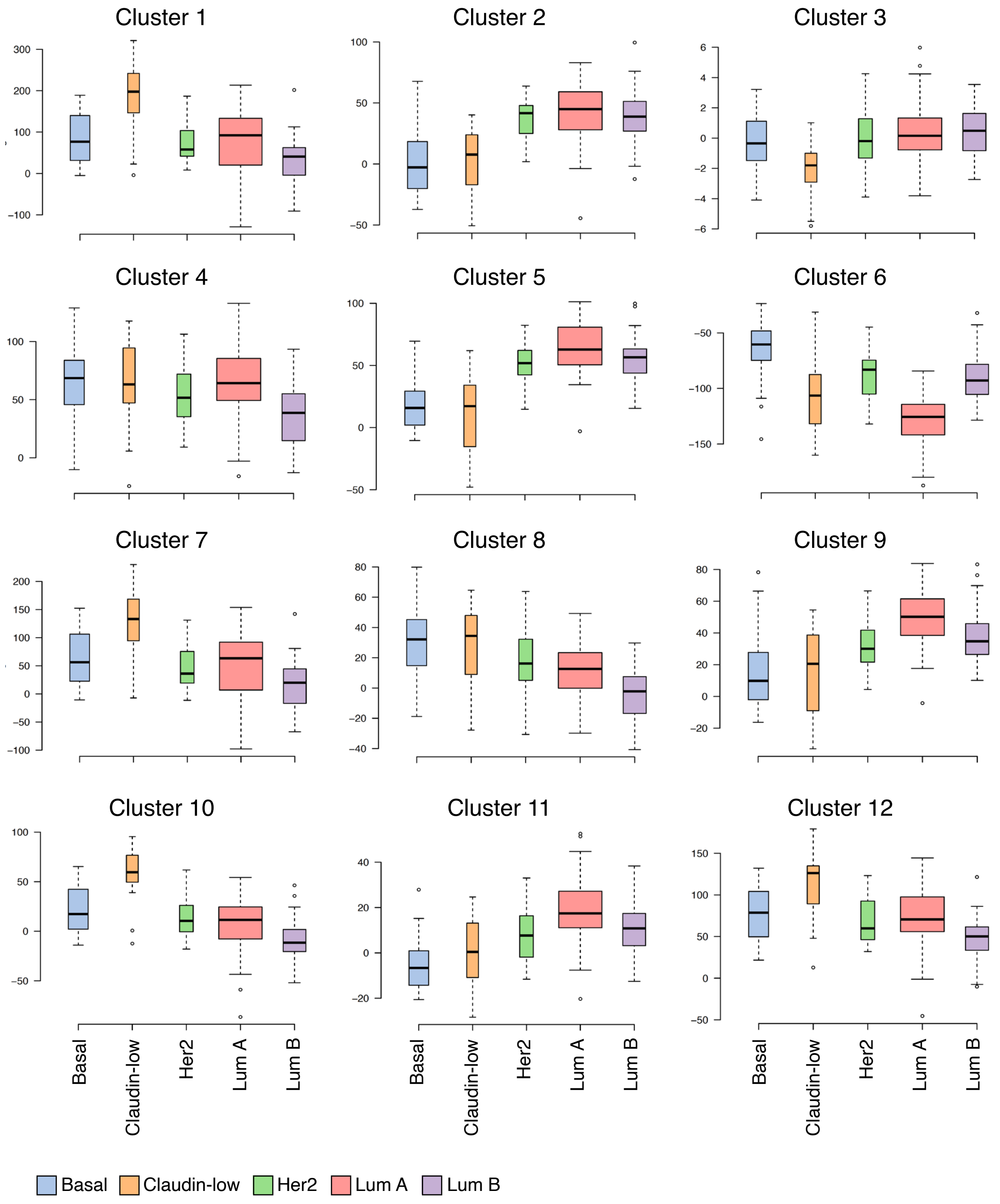

Figure S4. Relationship between cancer subtypes and normal epithelial subtypes during development. Boxplots of signature expression scores for each of the 12 clusters shown in Fig. 2d by human breast cancer subtype. Breast cancer expression data were obtained from GEO series GSE18229 [29] and processed as previously described [32]. Expression scores represent average log2-expression of the marker genes for each cluster.

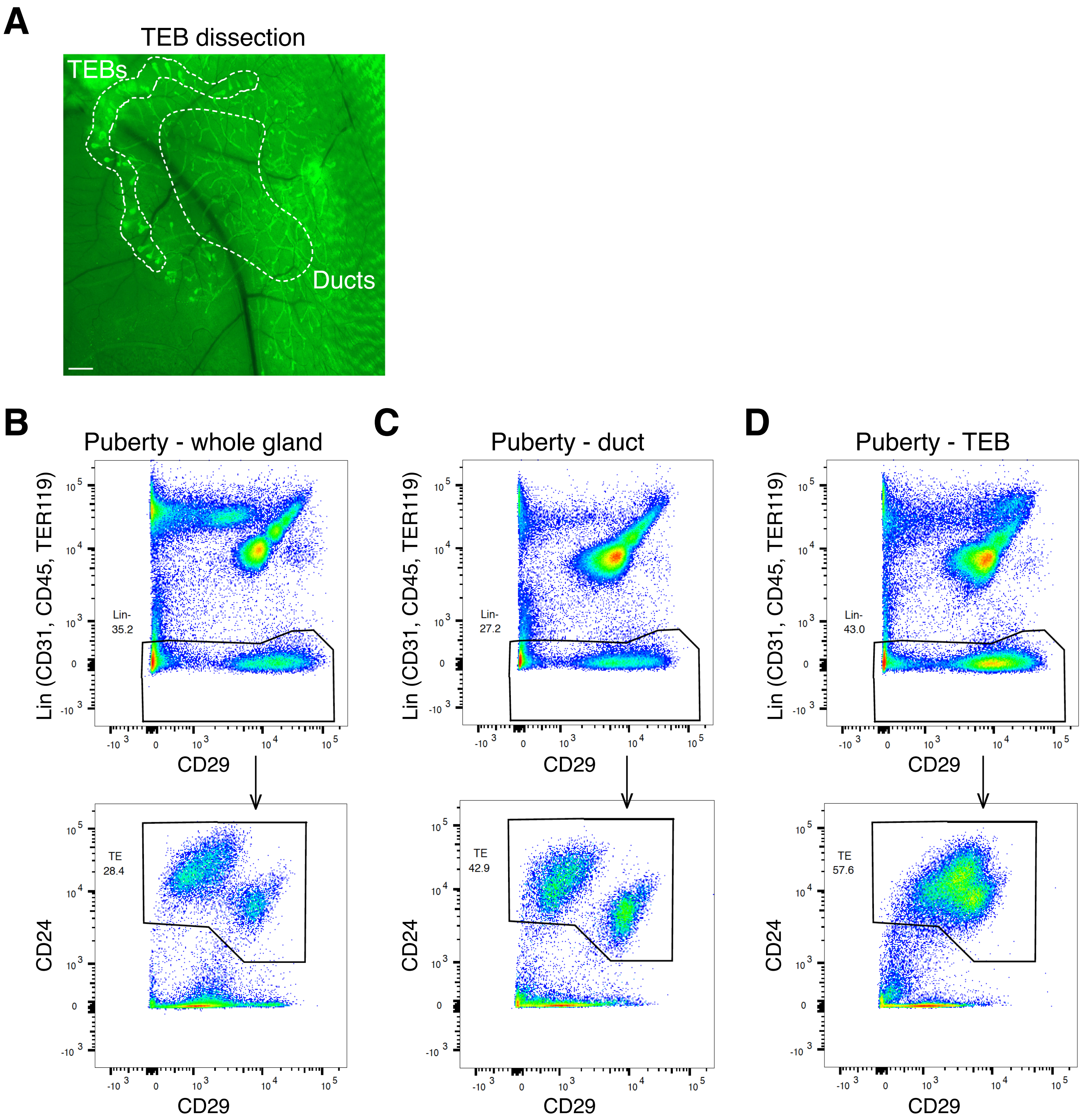

Figure S5. Cellular analysis of microdissected TEBs and ducts. (**A**) Image of whole third mammary gland, indicating dissection of TEBs and ducts from an *E-cadherin-GFP* mammary gland under fluorescence microscopy. (**B-D**) FACS analysis of 5 week-old mammary glands (B), microdissected ducts (C) and microdissected TEBs (D) from 5-week old *E-cadherin-GFP* mammary glands (n = 3 mice). Shown are live cells, gated on hematopoietic and endothelial lineage markers. Lineage-negative cells were sorted based on CD24 and CD29 expression using pubertal 5 week-old mammary glands to set gates.

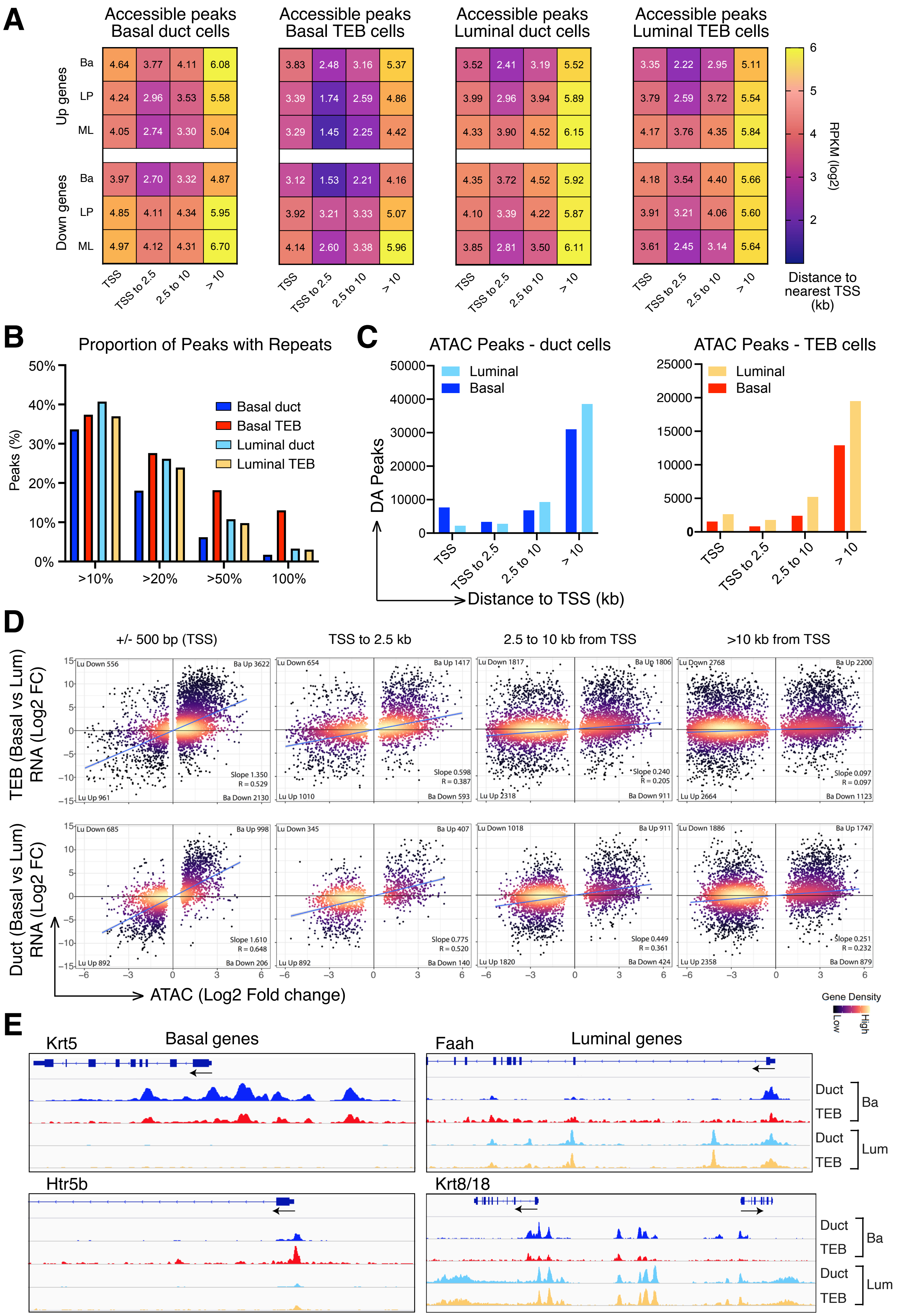

A
Accessible peaks Basal duct cells
Accessible peaks Basal TEB cells
Accessible peaks Luminal duct cells
Accessible peaks Luminal TEB cells
Up genes
Down genes
Ba
LP
ML
4.64 3.77 4.11 6.08
4.24 2.96 3.53 5.58
4.05 2.74 3.30 5.04
3.97 2.70 3.32 4.87
4.85 4.11 4.34 5.95
4.97 4.12 4.31 6.70
3.83 2.48 3.16 5.37
3.39 1.74 2.59 4.86
3.29 1.45 2.25 4.42
3.12 1.53 2.21 4.16
3.92 3.21 3.33 5.07
4.14 2.60 3.38 5.96
3.52 2.41 3.19 5.52
3.99 2.96 3.94 5.89
4.33 3.90 4.52 6.15
4.35 3.72 4.52 5.92
4.10 3.39 4.22 5.87
3.85 2.81 3.50 6.11
3.35 2.22 2.95 5.11
3.79 2.59 3.72 5.54
4.17 3.76 4.35 5.84
4.18 3.54 4.40 5.66
3.91 3.21 4.06 5.60
3.61 2.45 3.14 5.64
TSS
TSS to 2.5
2.5 to 10
>10
RPKM (log2)
Distance to nearest TSS (kb)
B
Proportion of Peaks with Repeats
Peaks (%)
Basal duct
Basal TEB
Luminal duct
Luminal TEB
>10%
>20%
>50%
100%
C
ATAC Peaks - duct cells
ATAC Peaks - TEB cells
Luminal
Basal
DA Peaks
TSS
TSS to 2.5
2.5 to 10
> 10
Distance to TSS (kb)
D
+/- 500 bp (TSS)
TSS to 2.5 kb
2.5 to 10 kb from TSS
>10 kb from TSS
TEB (Basal vs Lum) RNA (Log2 FC)
Duct (Basal vs Lum) RNA (Log2 FC)
Lu Down 556
Ba Up 3622
Slope 1.350
R = 0.529
Lu Up 961
Ba Down 2130
Lu Down 654
Ba Up 1417
Slope 0.598
R = 0.387
Lu Up 1010
Ba Down 593
Lu Down 1817
Ba Up 1806
Slope 0.240
R = 0.205
Lu Up 2318
Ba Down 911
Lu Down 2768
Ba Up 2200
Slope 0.097
R = 0.097
Lu Up 2664
Ba Down 1123
Lu Down 685
Ba Up 998
Slope 1.610
R = 0.648
Lu Up 892
Ba Down 206
Lu Down 345
Ba Up 407
Slope 0.775
R = 0.520
Lu Up 892
Ba Down 140
Lu Down 1018
Ba Up 911
Slope 0.449
R = 0.361
Lu Up 1820
Ba Down 424
Lu Down 1886
Ba Up 1747
Slope 0.251
R = 0.232
Lu Up 2358
Ba Down 879
ATAC (Log2 Fold change)
Gene Density
Low
High
E
Basal genes
Luminal genes
Krt5
Faah
Htr5b
Krt8/18
Duct
TEB
Ba
Lum

Figure S6. Differential accessible peaks identified from ATAC-sequencing analysis of dissected TEBs and ducts. (**A**) ATAC-seq coverage for peaks associated with the adult mammary epithelial signature genes for Basal, LP and ML cells. MACS peaks are classified based on their distance to the signature gene TSS. Values are the mean log2 RPKM+1 for all genes within each signature, inclusive of all peaks within each categorized region, reads not found within a peak were not included. (**B**) Proportion of ATAC-seq peaks that overlap with known repeat elements, based on >10%, >20%, >50% or 100% overlap between the peak and a repeat element; note genomic repeat coverage is ~40%. (**C**) The total number of DA peaks between basal and luminal duct cells and basal and luminal TEB cells. Peaks are categorized based on distance to their nearest TSS. (**D**) Scatterplots of gene expression changes (log2 RNA-seq fold change (FC)) versus differences in accessibility of its nearest DA peak (log2 ATAC-seq fold change), categorized based on the peak distance to the nearest TSS. Shown are only those genes associated with the DA peaks generated in each comparison. Top panels are the comparisons of basal TEB vs luminal TEB cells and the bottom panels are the basal duct cells versus the luminal duct cells. Displayed on each graph is the linear regression with standard deviation and slope, R correlation values and the genes within each quadrant. (**E**) Snapshots of ATAC-seq coverage for genes with TEB- or Duct-specific enrichment and examples of acquired proximal accessibility in luminal-specific genes.

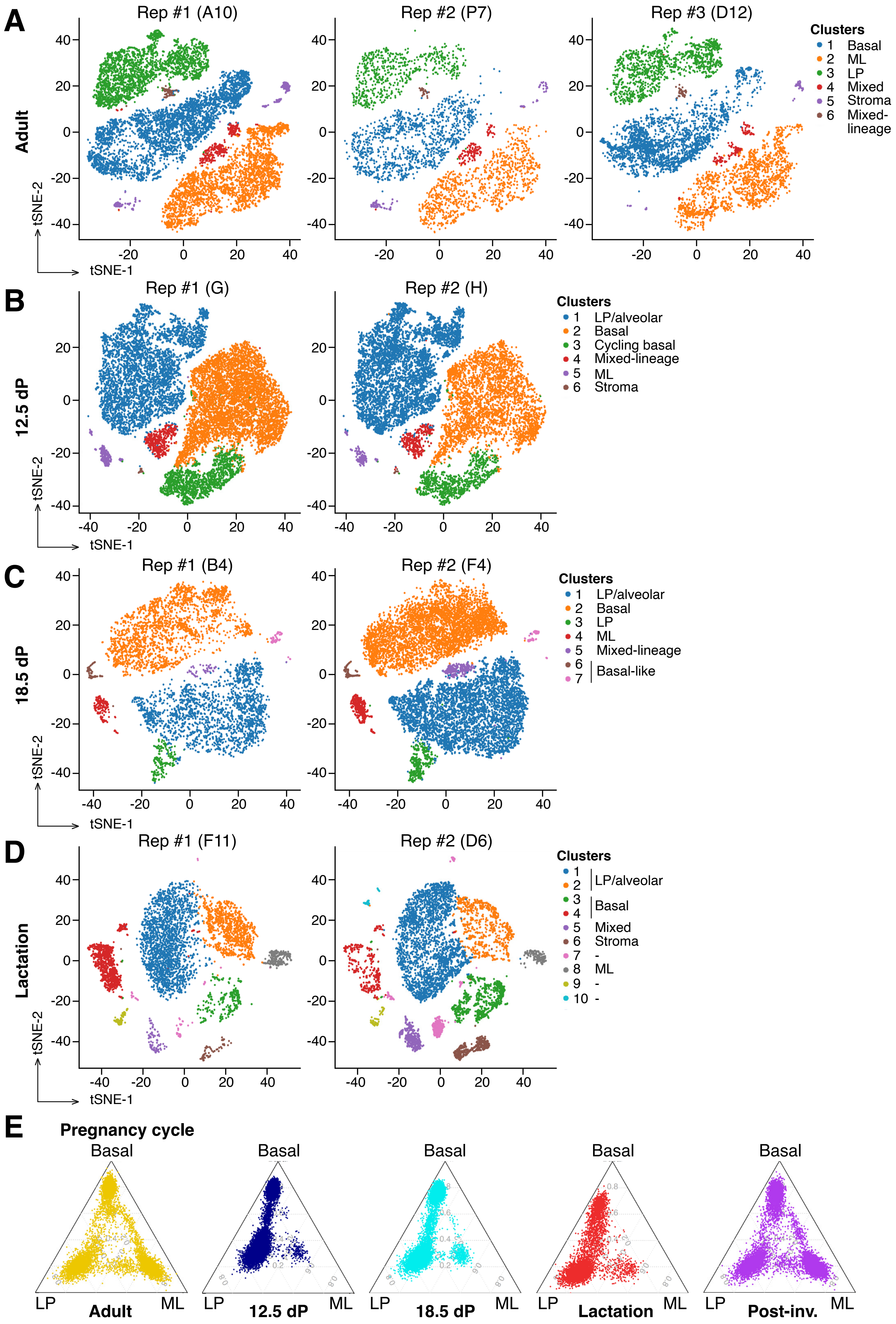
A
Rep #1 (A10)
Rep #2 (P7)
Rep #3 (D12)
Adult
Clusters
1 Basal
2 ML
3 LP
4 Mixed
5 Stroma
6 Mixed-lineage
tSNE-1
tSNE-2
B
Rep #1 (G)
Rep #2 (H)
12.5 dP
Clusters
1 LP/alveolar
2 Basal
3 Cycling basal
4 Mixed-lineage
5 ML
6 Stroma
C
Rep #1 (B4)
Rep #2 (F4)
18.5 dP
Clusters
1 LP/alveolar
2 Basal
3 LP
4 ML
5 Mixed-lineage
6
7 Basal-like
D
Rep #1 (F11)
Rep #2 (D6)
Lactation
Clusters
1
2 LP/alveolar
3
4 Basal
5 Mixed
6 Stroma
7 -
8 ML
9 -
10 -
E
Pregnancy cycle
Basal
LP
ML
Adult
12.5 dP
18.5 dP
Lactation
Post-inv.

Figure S7. (**A-D**) Replicate biological samples for adult (A), 12.5 days pregnancy (B), 18.5 days pregnancy (C) and 10 days lactation (D). At each developmental stage, the replicate scRNA-seq profiles have been integrated and clustered, then depicted as separate t-SNE plots for each replicate. All samples were taken from FVB/N mice (Table 1) (n=3-4 mice for adult, n=2 mice for other stages). The lineage identity of each cluster was identified by lineage expression signatures. Cluster 4 in (D) likely represents stellate myoepithelial cells in lactation, based on expression analysis. (**E**) Same as Fig. 6a but separated into separate ternary plots for each pregnancy cycle stage.

Table S1. scRNA-seq quality control statistics.

		Before cell filtering									Cell filtering				
					Reads per cell			Genes per cell				NGenes limits			
**Label**	**Description**	**NCells**	**NReads**	**NGenes**	**Min**	**Median**	**Max**	**Min**	**Median**	**Max**	**Mt limit**	**Lower**	**Upper**	**NReads Limit**	**NCells after Filtering**
E18-ME	Embryonic mammary	6969	54173813	19757	500	6969	52606	102	2082	6189	0.15	500	5000	30000	6398
E18-Skin	Embryonic adjacent skin	9184	50904927	20189	501	5220.5	25912	100	1837	4851	0.15	500	4500	20000	9025
Pre-D5-BL6	Early postnatal	3886	42821531	19699	506	9409.5	70970	34	2743	7508	0.2	500	6000	40000	3728
Pre-BL6	Pre-puberty	4183	39445446	19287	505	7225	76720	22	2376	8350	0.2	500	6000	35000	4037
TEB	Puberty TEBs	2087	45688910	18298	503	20652	91847	50	4260	7801	0.15	500	7000	55000	1847
Duct	Pubery Ducts	1973	29525671	17892	502	12673	83864	44	3096	7615	0.15	500	6500	50000	1700
Adult-FVB-A10	Adult FVB/N (A10)	11936	69643560	19964	500	4906	44959	39	1574	5805	0.2	500	3500	17000	10710
Adult-FVB-P7	Adult FVB/N (P7)	3481	9920346	16177	502	2472	13686	196	1092	3328	0.15	500	2250	7000	3187
Adult-FVB-D12	Adult FVB/N (D12)	5713	34121281	18741	504	4940	34346	90	1734	5515	0.2	500	4500	25000	5576
Adult-BL6	Adult C57BL/6	13269	71021911	20362	500	4721	29405	37	1811	5393	0.2	500	4500	22000	12525
Adult-SW	Adult SWISS	11135	85865973	20611	503	6611	64210	36	2128	7186	0.2	500	6000	35000	10319
Preg-D12-H	Pregnancy 12.5 days (H)	11406	66274512	18836	501	5058	29284	48	1621	4692	0.2	500	4000	20000	11120
Preg-D12-G	Pregnancy 12.5 days (G)	13877	71602020	19140	502	4118	30312	42	1492	4702	0.2	500	4000	20000	13397
Preg-D18-F4	Pregnancy 18.5 days (F4)	12510	56653320	18695	502	3514	23986	59	1213	3629	0.2	500	3000	18000	11847
Preg-D18-B4	Pregnancy 18.5 days (B4)	5696	42983751	18531	503	5168	41237	31	1644	5192	0.2	500	4000	28000	5075
Lac-D10-F11	Lactation (F11)	10877	51526155	17504	795	2109	49288	53	495	3005	0.2	500	2500	30000	5287
Lac-D10-D6	Lactation (D6)	9878	93234616	19127	500	4065	73768	26	960	4271	0.25	500	3000	40000	6804
PI-W3	Post-involution	11311	54003067	19734	500	3734	46771	47	1378	5753	0.2	500	4000	25000	10017
	**Total**	**149371**													**132599**
